# FOXQ1 Regulates Brain Endothelial Mitochondrial Function by Orchestrating Calcium Signaling and Cristae Morphology

**DOI:** 10.1002/advs.202503082

**Published:** 2025-08-30

**Authors:** Wenzheng Zou, Yuqing Lv, Lin Li, Shukui Zhang, Jiaqi Liang, Chengchao Wu, Enyu Huang, Jianwei Jiao, Jingjing Zhang

**Affiliations:** ^1^ Zhanjiang Key Laboratory of Zebrafish Model for Development and Disease Affiliated Hospital of Guangdong Medical University Zhanjiang 523710 China; ^2^ Key Laboratory of Organ Regeneration and Reconstruction State Key Laboratory of Stem Cell and Reproductive Biology Institute of Zoology Chinese Academy of Sciences Beijing 100101 China; ^3^ University of Chinese Academy of Sciences Beijing 100049 China; ^4^ Beijing Institute for Stem Cell and Regenerative Medicine Institute for Stem Cell and Regeneration Chinese Academy of Sciences Beijing 100101 China; ^5^ School of Medical Technology Guangdong Medical University Dongguan 523808 China

**Keywords:** brain endothelial cells, calcium signaling, cristae organization, FOXQ1, mitochondrial metabolism

## Abstract

The blood‐brain barrier (BBB) maintains brain homeostasis through specialized functions including tight junction formation and selective transport of brain endothelial cells (ECs). While ECs are generally thought to rely primarily on glycolysis for energy production, the transcriptional mechanisms underlying their metabolic specialization in the brain endothelium remain poorly understood, especially considering the brain's extraordinary energy demands. Through comparative transcriptomic analysis, it is demonstrated that brain endothelial cells are enriched for mitochondrial function genes, with forkhead box protein 1 (FOXQ1) being selectively expressed in cerebral vasculature. Conditional knockout of *Foxq1* in endothelial cells results in severe mitochondrial dysfunction, including disrupted cristae morphology, reduced oxygen consumption, and impaired adenosine triphosphate (ATP) production. Mechanistically, FOXQ1 directly regulates two key pathways: calcium signaling through huntingtin‐associated protein (HAP1)‐mediated endoplasmic reticulum (ER)‐mitochondrial calcium transfer, and mitochondrial structural integrity via AarF domain‐containing protein kinase 1 (ADCK1)‐dependent cristae organization. These findings reveal that brain endothelial cells rely on oxidative phosphorylation rather than glycolysis alone, challenging the prevailing metabolic paradigm for endothelial cells. This work establishes FOXQ1 as an important regulator of brain endothelial metabolism and provides new insights into the molecular basis of cerebrovascular specialization, with implications for understanding vascular dysfunction in neurological diseases.

## Graphical abstract

FOXQ1 emerges as a master transcriptional regulator of brain endothelial metabolism, orchestrating mitochondrial function through dual control of calcium signaling and cristae organization. This study reveals that brain endothelial cells rely on oxidative phosphorylation rather than glycolysis alone, challenging the current metabolic paradigm and providing new mechanistic insights into cerebrovascular specialization with important implications for neurological diseases.

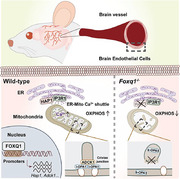

## 1 Introduction

The vascular endothelium lines the entire circulatory system. It is a dynamic, adaptable interface that varies functionally across organs.^[^ ^1^ ^]^ Endothelial cells (ECs) adopt tissue‐specific phenotypes to meet the physiological demands of their local microenvironments. In the central nervous system (CNS), endothelial specialization is exemplified by the formation of the blood‐brain barrier (BBB), where continuous tight junctions restrict molecule diffusion and tightly regulate substance transport into the brain.^[^ ^2^ ^]^ The BBB plays a critical role in maintaining brain homeostasis by allowing the passage of essential nutrients while preventing the entry of neurotoxic substances and pathogens.

While the structural and molecular components of the BBB, including tight junction proteins and specialized transporters, have been well characterized, much less is known about the metabolic adaptations that enable cerebral ECs to maintain these specialized functions. A prevailing view holds that ECs rely primarily on anaerobic glycolysis, which supplies over 95% of their adenosine triphosphate (ATP), especially under hypoxic conditions such as those encountered during angiogenesis.^[^ ^3^ ^]^ This glycolytic bias is thought to minimize oxygen consumption and allow better oxygen delivery to surrounding tissues. However, this model may not fully apply to brain ECs.

Despite comprising only ≈2% of body mass, the brain consumes approximately 20% of the body's oxygen and 25% of its circulating glucose.^[^ ^4^ ^]^ These exceptionally high metabolic demands impose unique energetic constraints on the cells forming the BBB, suggesting that cerebral ECs may require more robust energy‐producing mechanisms to support both barrier integrity and active transport. Consistent with this, ultrastructural studies have revealed that brain ECs contain a disproportionately high mitochondrial volume, accounting for 8–11% of the cytoplasmic space, which is approximately 2 to 5 times greater than that of peripheral ECs.^[^ ^5^ ^]^ This observation raises the possibility that oxidative phosphorylation (OXPHOS), rather than glycolysis alone, plays a more prominent role in sustaining the functions of brain ECs.

To investigate this possibility, we performed comparative transcriptomic analyses of ECs across multiple organs. Our data revealed that brain ECs are transcriptionally enriched for genes associated with mitochondrial function and oxidative metabolism, pointing to a specialized metabolic program that may be critical for BBB function. To understand the regulatory basis of this metabolic specialization, we examined transcription factors enriched in brain ECs. Prior studies have identified several brain EC‐enriched transcription factors, such as FOXF2, ZIC3, and LEF1, downstream of Wnt/β‐catenin signaling, which are known to regulate BBB development and junction formation.^[^ ^6^ ^]^ However, the role of FOXQ1, another Wnt‐responsive transcription factor selectively expressed in brain ECs, has not been previously explored in this context. In this study, we identified FOXQ1 as a key regulator of mitochondrial function in cerebral ECs. We show that FOXQ1 promotes mitochondrial homeostasis, enhances OXPHOS activity, and modulates calcium signaling and cristae morphology, thereby supporting the high energy demands of the BBB.

These findings not only expand the current repertoire of BBB‐regulatory transcription factors but also offer novel mechanistic insights into the metabolic adaptations of brain ECs. As cerebral vascular dysfunction is involved in various neurological diseases, including stroke, vascular dementia, and Alzheimer's disease,^[^ ^7^ ^]^ elucidating the metabolic characteristics of brain ECs could advance our understanding of vascular heterogeneity and inform potential therapeutic strategies.

## 2 Results

### 2.1 Inter‐Tissue Heterogeneity of Brain Endothelial Cells

**Figure 1:**
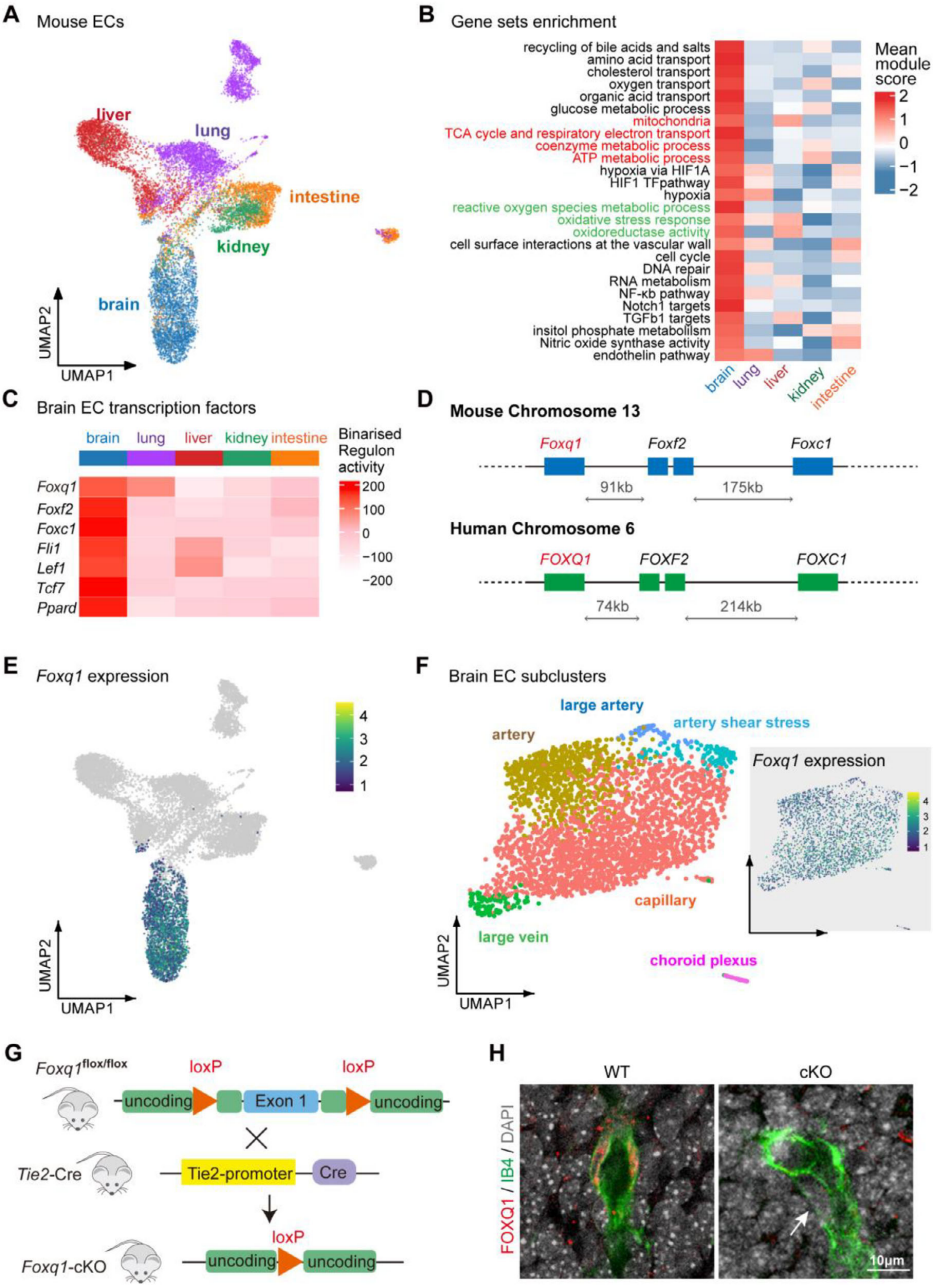
Tissue‐specific characteristics of brain endothelial cells in mice and development of endothelial‐specific *Foxq1* knockout mice. A) Uniform Manifold Approximation and Projection (UMAP) plot of endothelial cells (ECs), color coded for tissue types: brain, liver, lung, kidney, and intestine.^[^ ^6c^ ^]^ B) Heat map showing processes enriched in brain ECs relative to those in liver, lung, kidney, and intestine. C) Single‐cell regulatory network inference and clustering (SCENIC) analysis to predict transcription factors predominant in brain ECs. D) Genetic map for the arrangement and relative positions of *Foxq1*, *Foxf2*, and *Foxc1* on chromosome 13 in mice and chromosome 6 in humans. E) Normalized expression of *Foxq1* in UMAP plot. F) (Left) UMAP plot visualization of brain EC subclusters. (Right) Normalized expression of *Foxq1* in these brain ECs. G) Schematic diagram of generation of the Tie2‐cre‐induced *Foxq1* conditional knockout (cKO) mouse using a CRISPR‐Cas9‐mediated double‐nicking strategy. Notably, the *Foxq1* gene consists of a single exon. H) Representative images from E16 wild‐type (WT) and *Foxq1* cKO brain blood vessels, stained for FOXQ1 (red) and Isolectin B4 (IB4, green), and with DAPI (grey). An arrow indicates the absence of the FOXQ1 immunofluorescence signal in the brain ECs of *Foxq1* cKO mice. Scale bar, 10 µm.

To explore transcriptional differences between brain endothelial cells (ECs) and those from other tissues, we utilized single‐cell RNA sequencing data from mouse vascular ECs across five tissues‐brain, liver, lung, kidney, and intestine‐as reported by Kalucka et al.^[^ ^6c^ ^]^ Uniform manifold approximation and projection (UMAP) analyses indicated that while ECs from the liver, lung, kidney, and intestine exhibited relative similar transcriptional profiles, brain ECs displayed more unique characteristics (**Figure** **1**A). Functional clustering analysis of brain EC‐specific genes identified sets associated with substance transport, aligning with the known low permeability of the blood‐brain barrier (BBB), which predominantly relies on active transport mechanisms for nutrient and waste exchange (Figure 1B and Table S1, Supporting Information). Notably, pathways related to mitochondria and energy metabolism were prominently represented (Figure 1B). Although it is well established that ECs primarily rely on glycolysis for ATP production,^[^ ^8^ ^]^ our analysis indicated that brain ECs engage in significant mitochondrial energy metabolic activity. Given the high metabolic demands of the brain,^[^ ^4^, ^9^ ^]^ this reflects the high energy demands of cerebral vasculature.

To understand the transcriptional regulatory networks, we investigated specific transcription factors in brain ECs. We identified several potential key regulators, including *Foxq1*, *Foxf2*, *Foxc1*, *Fli1*, *Lef1*, *Tcf7*, and *Ppard* (Figure 1C). Among which, *Foxq1*, *Foxf2*, and *Foxc1* are members of the forkhead‐box (FOX) family of transcription factors. Their adjacency on chromosomes in both mice and humans may facilitate coordinated gene transcription within a gene‐loop conformation (Figure 1D). Previous studies have shown that deletion of the *Foxf2* gene leads to intracranial hemorrhage and a leaky BBB,^[^ ^10^ ^]^ and that Foxc1 is essential for retinal angiogenesis and blood‐retina barrier formation,^[^ ^11^ ^]^ highlighting the critical roles of these FOX‐family transcription factors in maintaining BBB integrity. However, the function of FOXQ1 remains to be understood.

UMAP analysis indicated a specific expression of *Foxq1* in brain ECs (Figure 1E). We further categorized brain ECs into arterial, venous, and capillary subpopulations, observing that *Foxq1* expression was not confined to any specific subpopulation (Figure 1F). To determine whether FOXQ1 connects the unique mitochondrial and energy metabolism in brain ECs, we developed a model of endothelial *Foxq1* conditional knockout mice. Utilizing CRISPR technology, we engineered *Foxq1*‐flox mice and crossed them with Tie2‐cre mice, which express CRE recombinase specifically in vascular ECs (Figure 1G). This genetic strategy enabled the precise deletion of *Foxq1* in these cells. Immunofluorescence staining was performed to verify the effective knockout of *Foxq1* in brain ECs (Figure 1H).

### 2.2 Removal of FOXQ1 Leads to Mitochondrial Impairment in Mouse Brain Endothelial Cells

**Figure 2:**
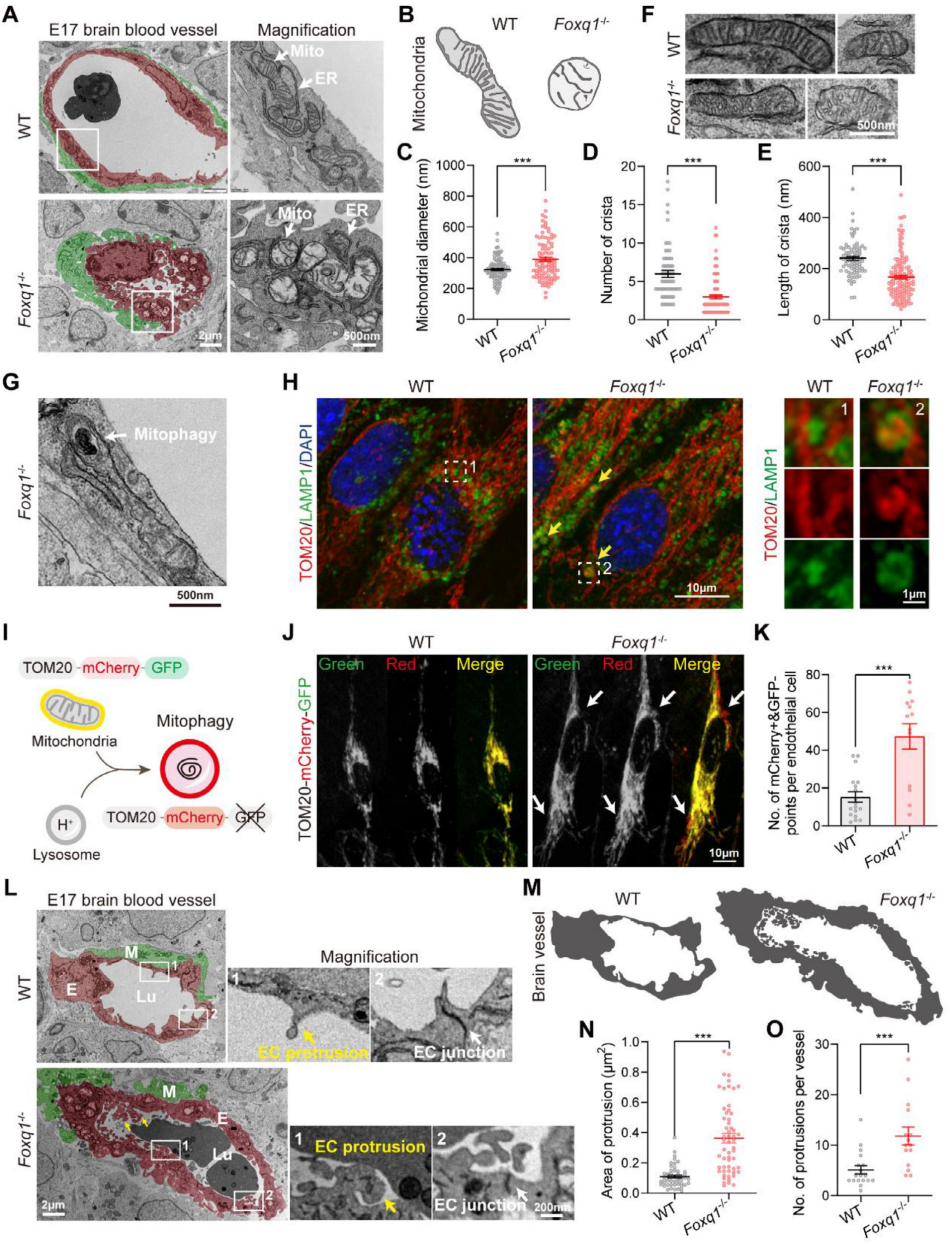
Impact of FOXQ1 deletion on brain vessel and mitochondrial morphology in brain ECs. A) TEM images showing the E17 wild‐type versus *Foxq1* cKO brain blood vessels (left) and magnified views (right) revealing the mitochondria and the endoplasmic reticulum (ER) in endothelial cells. Scale bars, 2 µm and 500 nm. Red color indicates endothelial cells and green color indicates muller cells. B) Schematic representative of mitochondrial morphology in E17 wild‐type and *Foxq1* cKO cells. C) Quantification of the mitochondrial diameter (*n* = 84 in wild‐type and 139 in *Foxq1* cKO from two independent experiments). D) Quantification of the number of mitochondrial crista (*n* = 64 mitochondria in wild‐type and 90 mitochondria in *Foxq1* cKO from two independent experiments). E) Quantification of the length of mitochondrial crista length (*n* = 103 crista in WT, *n* = 97 crista in cKO from two independent experiments). F) Additional representative TEM images of the elongated‐shaped mitochondria in the *Foxq1* cKO brain blood vessel. Scale bar, 500 nm. G) Representative TEM image showcasing mitophagy in *Foxq1* cKO brain EC. Scale bar, 500 nm. H) Immunofluorescence images of primary brain ECs from wild‐type and *Foxq1* cKO stained with TOM20 (red), LAMP1 (green), and DAPI (blue) (left). Magnified images show lysosomes engulfing mitochondrial fragments (right). Scale bar, 10 µm and 1 µm. I) Schematic of fluorescent tandem reporter for mitophagy. TOM20‐mCherry‐GFP is a pH‐sensitive sensor that emits both red and green fluorescence in mitochondria and emits only red fluorescence after their fusion with lysosomes due to GFP denaturation. J) Representative images of primary brain ECs from wild type and *Foxq1* cKO transfected with TOM20‐mCherry‐GFP lentivirus. White arrows indicate mitophagy in *Foxq1* cKO EC. Scale bar, 10 µm. K) Quantification of mCherry^+^GFP^−^ spots in primary ECs (*n* = 18 cells in WT and 13 cells in cKO). L) Representative TEM images of the E17 wild‐type and *Foxq1* cKO brain blood vessel. Scale bar, 2 µm. Magnification images highlight the EC protrusion (yellow arrows) and EC junction (white arrow). Scale bar, 200 nm. Red color indicates endothelial cells and green color indicates muller cells. M) Schematic diagram of wild‐type and *Foxq1* cKO brain blood vessel. N) Quantification of the protrusion area extending into the vascular lumen (*n* = 50 in wild‐type and 57 in *Foxq1* cKO from two independent experiments). O) Quantification of the number of protrusions per vessel (*n* = 18 in wild‐type and 15 in *Foxq1* cKO from two independent experiments). All data are shown as mean ± s.e.m. Statistical significance is noted as ***P < 0.001 using a two‐tailed unpaired Student's *t*‐test.

Interestingly, our examination of mitochondrial morphology in brain ECs from *Foxq1* knockout mice revealed significant defects. Transmission electron microscopy (TEM) suggested that in wild‐type brain ECs, mitochondria displayed a rod‐like shape with orderly, parallel cristae (**Figure** **2**A,B). In contrast, cKO cells exhibited enlarged mitochondria (Figure 2A–C), with a significant reduction in both the density and length of cristae (Figure 2D,E). Some mitochondria with mild defects retained their rod‐like shape; however, the cristae detached from the inner mitochondrial membrane, appearing vesicular or swollen with irregular arrangements (Figure 2F and Figure S1A–C, Supporting Information). Narrow cristae are known to enhance ATP synthesis through optimal enzyme organization, while vesicular cristae may reduce local flux, indicating functional impairment.^[^ ^12^ ^]^ These abnormalities likely contribute to the observed mitochondrial damage and subsequent induction of mitophagy (Figure 2G). Enhanced mitophagy was confirmed through immunostaining of primary brain EC cultures using TOM20 (Translocase of Outer Mitochondrial Membrane 20) and LAMP1 (Lysosome‐Associated Membrane Glycoprotein 1), revealing increased lysosome‐encapsulated mitochondria in *Foxq1* knockout cells compared to wild‐type cells (Figure 2H). Quantification using the *Tom20‐mCherry‐GFP* fusion construct, which indicates the quenching of GFP fluorescence in the acidic environment of lysosomes, further revealed a marked increase in mitophagy levels in *Foxq1* knockout brain endothelial cells (Figure 2I–K).

Additionally, TEM revealed marked irregularities in the vascular lumen of *Foxq1*‐deficient ECs, characterized by an abundance of protrusions, commonly referred to as “microvilli,” extending into the vascular lumen (Figure 2L–O). Microvilli are fewer in wild‐type vessels, where they are primarily localized at EC junctions, and are believed to sense blood flow and create resistance,^[^ ^13^ ^]^ potentially obstructing circulation (Figure 2L,M). In pathological conditions, such as cerebral ischemia, the number of microvilli significantly increases.^[^ ^14^ ^]^ In *Foxq1* knockout cells, the abundance of microvilli was consistently associated with mitochondrial swelling (Figure 2L,M). Of note, cells with only mild mitochondrial defects showed no significant change in microvilli numbers, suggesting a connection between increased microvilli and mitochondrial disorders (Figure S1D,E, Supporting Information).

### 2.3 *Foxq1* Deletion Preserves Vascular Development But Impairs Neurological Function

Despite the significant cellular defects observed at the mitochondrial level, we found that *Foxq1* deletion did not significantly impair overall vascular development or structural integrity. Isolectin‐B4 (IB4) immunostaining of embryonic day 16 (E16) brain sections demonstrated that key morphometric parameters of the developing cerebral vasculature, including vascular density and vessel diameter, remained unaltered in *Foxq1* knockout mice (Figure S2A–C, Supporting Information). Furthermore, the neurovascular unit architecture appeared normal, with intact vascular basement membrane integrity, preserved pericyte coverage, and normal astrocytic end‐foot association (Figure S2D–F, Supporting Information), indicating that blood‐brain barrier (BBB) structural components develop appropriately despite *Foxq1* absence.

To determine whether normal structural development translates to functional BBB integrity, we assessed barrier permeability using Evans blue extravasation assays. Intravenous injection of 2% Evans blue dye revealed no significant tracer extravasation in *Foxq1* cKO brains compared to wild‐type controls (Figure S2G,H, Supporting Information), confirming that BBB permeability remains intact under physiological conditions. Additionally, in vitro experiments demonstrated that migration, proliferation, and tube formation capabilities of *Foxq1*‐depleted primary brain endothelial cells were comparable to wild‐type cells (Figure S2I–P, Supporting Information), suggesting that basic angiogenic functions are preserved.

However, despite preserved vascular structure and barrier function, *Foxq1* cKO mice exhibited significant behavioral deficits that reflect compromised brain function. Motor coordination and balance were markedly impaired, as demonstrated by significantly shorter latency to fall in the Rotarod test compared to wild‐type controls (Figure S3A,B, Supporting Information). Cognitive function was similarly affected, with cKO mice spending less time in the target quadrant during the probe phase of the Morris Water Maze, indicating impaired spatial memory retention (Figure S3C,D, Supporting Information). In the open field test, cKO mice displayed altered exploration patterns, spending more time in the central zone while covering less total distance, suggesting diminished exploratory behavior and potential anxiety‐like responses (Figure S3E–G, Supporting Information).

Collectively, these findings demonstrate that while *Foxq1* deletion does not disrupt gross vascular architecture or barrier integrity, the mitochondrial dysfunction and cellular abnormalities in brain endothelial cells ultimately translate to compromised motor coordination, learning and memory, and cognitive function, highlighting the critical role of FOXQ1 in maintaining optimal brain endothelial cell function and overall neurological health.

### 2.4 *Foxq1* Deletion Led to Decreased Oxygen Consumption Rate and Mitochondrial Membrane Potential

**Figure 3:**
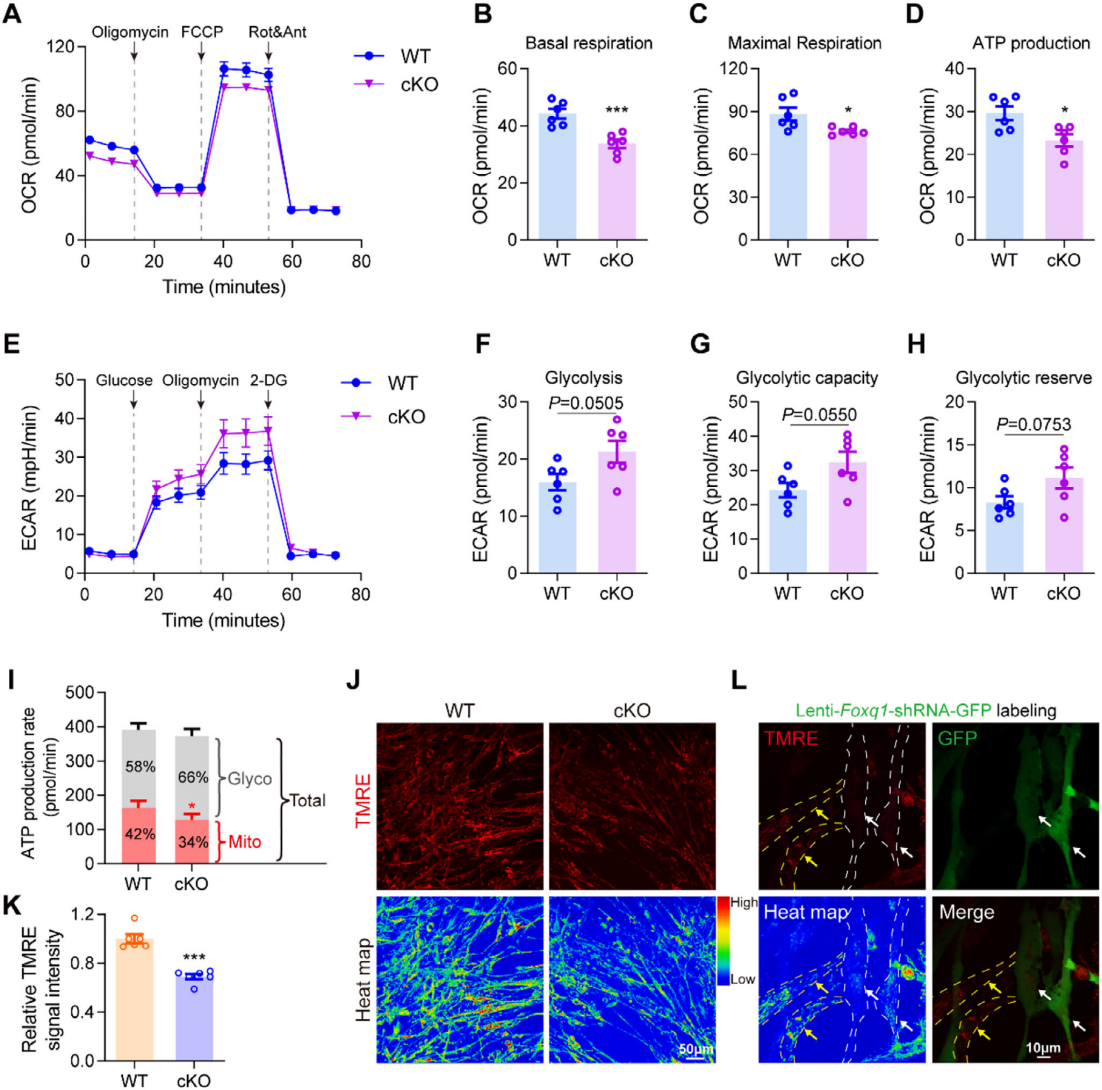
*Foxq1* deletion compromises cellular respiration and mitochondrial membrane potential in brain ECs. A) Oxygen consumption rate (OCR) measurements for wild type and *Foxq1* cKO primary brain ECs under basal conditions and following the sequential addition of oligomycin (1.5 µm), FCCP (0.5 µm), and rotenone (0.5 µm). B–D) Quantification of basal respiration, maximal respiratory capacity, and ATP production from 6 replications. E) Extracellular acidification rate (ECAR) levels for wild type and *Foxq1* cKO primary brain ECs under basal conditions and following the addition of glucose (10 mm), oligomycin (1 µm) and 2‐deoxy‐D‐glucose (2‐DG) (50 mm). F–H) Quantification of basal ECAR, glycolytic capacity, and glycolytic reserve from 6 replications. I) Quantification of ATP production rate from 6 replications. J) Representative images of the wild type and *Foxq1* cKO primary brain ECs staining with TMRE, a mitochondria membrane potential dye. Scale bar, 50 µm. K) Quantification of the relative mitochondria membrane potentials between wild type and *Foxq1* cKO primary brain ECs from 6 replications. L) Representative images of primary brain ECs infected with *Foxq1‐shRNA‐EF1a‐GFP* lentivirus and stained with TMRE. Yellow arrows indicate control ECs without infection, and the white arrows indicate ECs with FOXQ1 knockdown. Scale bar, 10 µm. Data are shown as A–H,K) mean ± s.e.m and L) mean + s.e.m. *P < 0.05, **P < 0.01, ***P < 0.001. Two‐tailed unpaired B–D,F–H,K) Students’ *t*‐test was used for, and one‐way ANOVA followed by L) Tukey's post‐hoc test.

Given the observed mitochondrial morphological abnormalities and enhanced mitophagy in *Foxq1*‐deficient ECs, we hypothesized that these structural alterations would translate into functional metabolic defects. To comprehensively assess mitochondrial bioenergetic capacity, we performed real‐time measurements of cellular metabolism using Seahorse XF technology in primary brain ECs.^[^ ^15^ ^]^ Analysis of oxygen consumption rate (OCR), the gold standard measure of mitochondrial respiratory activity, revealed profound bioenergetic dysfunction in *Foxq1* knockout cells. Both basal and maximal respiration rates were significantly reduced compared to wild‐type controls (**Figure** **3**A–D), indicating compromised electron transport chain capacity. Critically, oligomycin treatment demonstrated that ATP synthesis‐coupled oxygen consumption was markedly decreased, suggesting that the respiratory defect directly impairs primary energy production machinery.

In response to mitochondrial dysfunction, *Foxq1* knockout cells exhibited a characteristic metabolic reprogramming toward anaerobic metabolism. Extracellular acidification rate (ECAR) measurements revealed significantly elevated glycolytic activity (Figure 3E–H), indicating increased lactate production and proton efflux. Direct quantification of ATP production confirmed this metabolic insufficiency: while glycolytic ATP (glycoATP) production was indeed increased in *Foxq1* knockout cells, the dramatic reduction in mitochondrial ATP (mitoATP) production resulted in an overall deficit in total cellular ATP availability (Figure 3I).

To further characterize the mechanistic basis of respiratory impairment, we assessed mitochondrial membrane potential (ΔΨm) using tetramethylrhodamine ethyl ester (TMRE) fluorescence. Both genetic approaches—*Foxq1* cKO and lentiviral *Foxq1* knockdown—resulted in significant ΔΨm depolarization (Figure 3J–L). This finding provides direct evidence of electron transport chain (ETC) dysfunction, as membrane potential is generated by proton pumping across the inner mitochondrial membrane during oxidative phosphorylation. Collectively, these results establish FOXQ1 as a critical regulator of mitochondrial bioenergetics in brain endothelial cells.

### 2.5 FOXQ1 Regulates the Expression of Genes Associated with Mitochondrial Metabolism

Having established that FOXQ1 deficiency leads to profound mitochondrial dysfunction, we sought to elucidate the molecular mechanisms underlying this regulation. Given that several FOX family members, including FOXG1,^[^ ^16^ ^]^ FOXO1,^[^ ^17^ ^]^ FOXO3a,^[^ ^18^ ^]^ and FOXM1,^[^ ^19^ ^]^ are known to localize to mitochondria where they perform organelle‐specific functions, we first investigated FOXQ1's subcellular distribution. Subcellular localization studies using a FOXQ1‐GFP fusion construct in primary brain endothelial cells revealed exclusive nuclear localization, with no detectable mitochondrial signal (Figure S4A, Supporting Information). This nuclear restriction was further supported by bioinformatics analysis, which confirmed the absence of mitochondrial targeting sequences in the FOXQ1 protein. These findings indicate that FOXQ1 regulates mitochondrial function indirectly through nuclear transcriptional control rather than direct organellar targeting.

**Figure 4:**
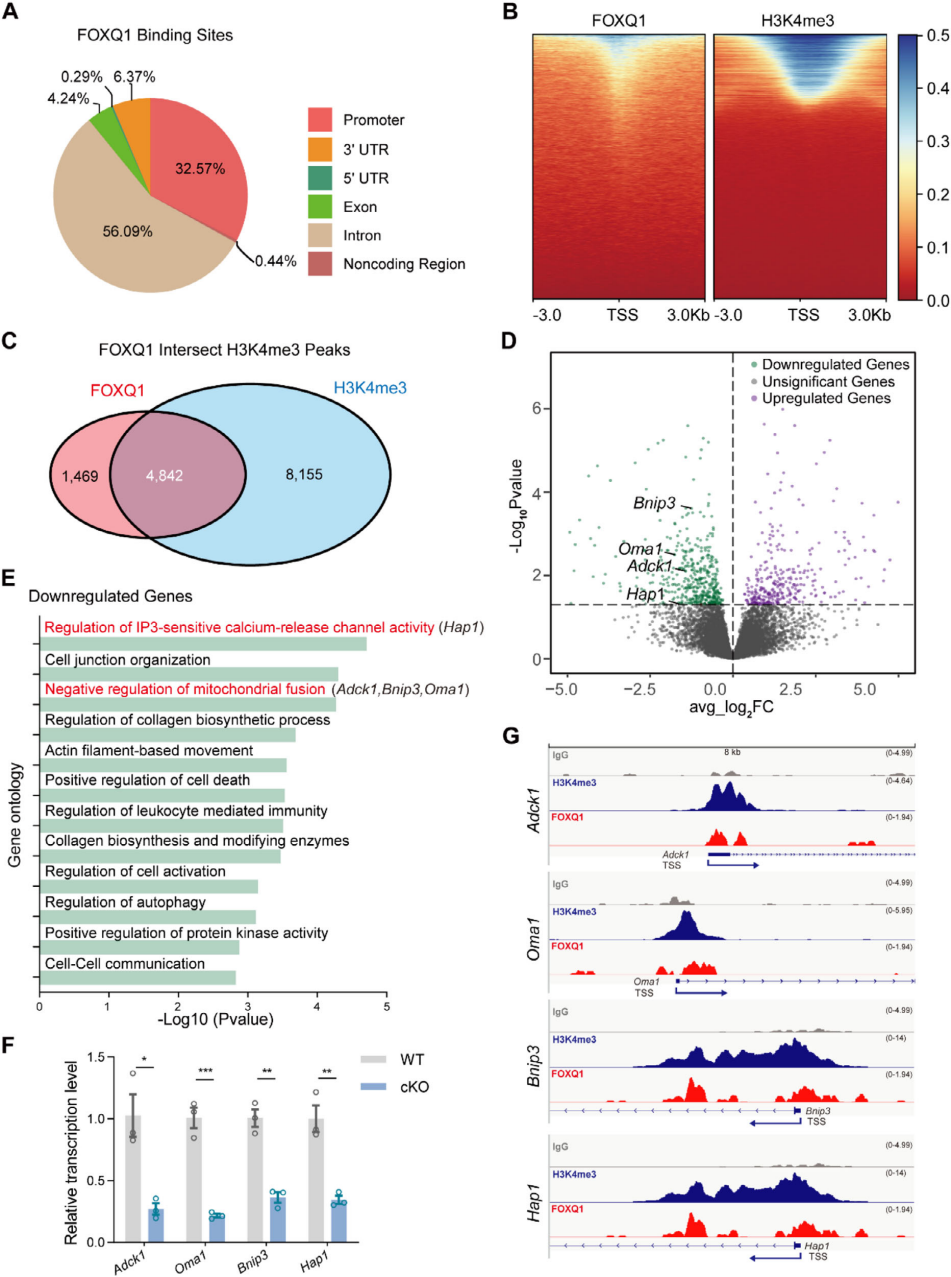
FOXQ1 binds to H3K4me3 active promoters and regulates mitochondria associated genes in brain ECs. A) Pie chart plot for the genomic distribution of FOXQ1 binding sites in primary brain ECs. B) Heat maps for the localization of FOXQ1 and H3K4me3 binding within ±3kb from the centers of their respective peaks. C) Venn diagram depicting the significant overlap between FOXQ1 and H3K4me3 binding sites in primary brain ECs. D) Volcano plots showing the downregulated (green) and upregulated (magenta) genes identified in the *Foxq1* cKO cells at E18 cerebral cortex in RNA‐seq data. Labels pinpoint representative mitochondria‐associated genes that are differentially expressed. E) Gene ontology (GO) enrichment analysis for downregulated genes identified in RNA‐seq data. F) Quantitative PCR (qPCR) analysis of *Adck1*, *Oma1*, *Bnip3*, and *Hap1* mRNA levels in wild type and *Foxq1* cKO primary brain ECs (*n* = 3 replications). G) Genomics occupancy profiles from CUT‐Tag analysis revealing distinct of FOXQ1 and H3K4me3 enrichments at the promoters of mitochondrial‐related genes *Adck1*, *Oma1*, *Bnip3*, and *Hap1*. Data are shown as mean ± s.e.m for (F). *P < 0.05, **P < 0.01, ***P < 0.001. Two‐tailed unpaired Students’ *t*‐test was used for (F).

To systematically identify FOXQ1's direct transcriptional targets, we performed Cleavage Under Targets and Tagmentation (CUT&Tag) using an HA‐tagged FOXQ1 construct.^[^ ^20^ ^]^ This high‐resolution chromatin profiling technique revealed that 32.57% of FOXQ1 binding events occurred within promoter regions (**Figure** **4**A), suggesting a primary role in transcriptional initiation rather than distal enhancer regulation. To distinguish between active and inactive promoter binding, we overlaid FOXQ1 CUT&Tag peaks with histone H3 lysine 4 trimethylation (H3K4me3) marks, which define transcriptionally active promoters.^[^ ^21^ ^]^ This analysis identified 4842 genes where FOXQ1 co‐localizes with H3K4me3 at their promoters (Figure 4B,C), indicating direct regulation of actively transcribed genes. Gene Ontology (GO) enrichment analysis of these co‐occupied genes revealed significant clustering in pathways related to oxidative stress response and mitochondrial processes (Figure S4B, Supporting Information), providing the first direct evidence linking FOXQ1 to mitochondrial gene regulation.

To assess the functional consequences of FOXQ1 binding, we performed Smart‐seq2‐based RNA sequencing on brain endothelial cells isolated from E18 wild‐type and *Foxq1* conditional knockout brains (Figure 4D). Pathway enrichment analysis of downregulated transcripts revealed significant enrichment in processes involving IP3‐sensitive calcium signaling and negative regulation of mitochondrial fusion (Figure 4E), both of which are critical for mitochondrial homeostasis and bioenergetics. Real‐time PCR validation confirmed decreased expression of key mitochondrial regulatory genes in *Foxq1* knockout cells, including *Adck1*, *Oma1*, *Bnip3*, and *Hap1* (Figure 4F). Importantly, Integrative Genomics Viewer (IGV) analysis demonstrated direct co‐localization of FOXQ1 and H3K4me3 signals at the promoters of these validated target genes (Figure 4G), establishing a direct mechanistic link between FOXQ1 binding and transcriptional regulation.

Notably, while GO analysis of FOXQ1‐H3K4me3 co‐occupied genes suggested enrichment in apoptotic signaling pathways, TUNEL staining revealed no significant increase in apoptosis in FOXQ1‐deficient brain ECs (Figure S4B–D, Supporting Information), indicating that the observed mitochondrial dysfunction occurs independently of cell death pathways.

To understand the molecular basis of FOXQ1's transcriptional specificity, we employed AlphaFold‐based structural prediction to model FOXQ1‐DNA interactions.^[^ ^22^ ^]^ The predicted structure revealed that residues 153‐169 within the forkhead domain form a recognition α‐helix that inserts into the major groove of target DNA sequences (Figure S5A, Supporting Information). Sequence analysis of FOXQ1 binding sites near the transcription start sites of validated targets (*Adck1* and *Hap1*) identified a strong preference for the GTTTC motif (Figure S5B, Supporting Information). Detailed structural modeling revealed specific amino acid‐nucleotide interactions: the imidazole ring of histidine 165 (H165) forms hydrogen bonds with adenine‐thymine base pairs, while the guanidinium group of arginine 164 (R164) establishes hydrogen bonds with guanine residues (Figure S5C–E, Supporting Information). These findings establish that FOXQ1 employs sequence‐specific recognition of GTTTC motifs near transcription start sites to directly regulate mitochondrial metabolism genes, providing a molecular framework for understanding how nuclear FOXQ1 controls mitochondrial bioenergetics in brain endothelial cells.

### 2.6 FOXQ1 Promotes Calcium Flux between the Endoplasmic Reticulum and Mitochondria by Modulating *Hap1* Expression

Among the FOXQ1 target genes identified through our integrated genomic analysis, *Hap1* (Huntingtin Associated Protein 1) emerged as a particularly compelling candidate for mediating mitochondrial dysfunction. Previous studies in neurons have demonstrated that HAP1 forms a functional complex with inositol 1,4,5‐trisphosphate receptor type I (IP3R1) and Huntingtin (Htt), serving as a critical regulator of calcium signaling from the endoplasmic reticulum (ER).^[^ ^23^ ^]^ Given the fundamental role of calcium in mitochondrial bioenergetics, we hypothesized that FOXQ1‐mediated regulation of *Hap1* expression might control mitochondrial function through modulation of inter‐organellar calcium transfer.

**Figure 5:**
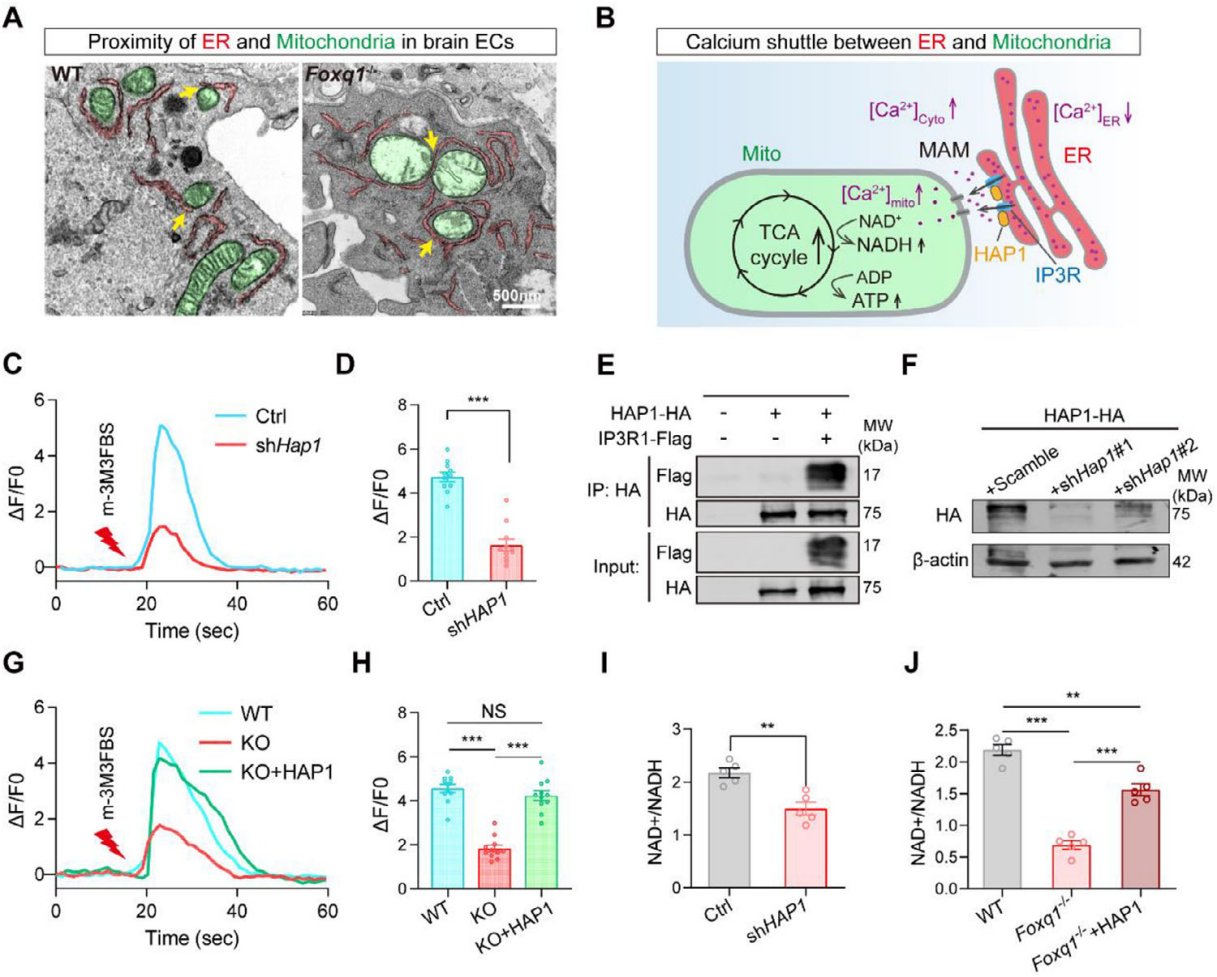
FOXQ1 promotes ER‐mitochondria calcium flux by regulating the expression of *Hap1*. A) Representative TEM images of wild type and *Foxq1* cKO brain ECs highlighting mitochondria‐associated membranes (MAMs) indicated by yellow arrows. Scale bar, 500 nm. B) Mechanism diagram for calcium transfer between ER and mitochondria. Activation of IP3R by the second messenger IP3 induces opening of the IP3R1‐HAP1‐Htt complex on the ER, releasing Ca^2+^ from the ER lumen and increasing Ca^2+^ concentration. The influx of Ca^2+^ into mitochondria influences mitochondrial enzyme activity and activates the electron transport chain, boosting NADH and ATP production. IP3R, inositol 1,4,5‐trisphosphate receptor; OMM, outer mitochondrial membrane; IMM, inner mitochondrial membrane; TCA, tricarboxylic acid cycle. C) Calcium transient traces in control brain ECs (blue line) and HAP1 knockdown brain ECs (red line) triggered by 20 µm m‐3M3FBS. D) Histogram depicting the average amplitudes of m‐3M3FBS triggered Ca^2+^ transients, represented by Δ*F*/*F*0 of Fluo‐4 (*n* = 11 cells from two independent experiments). E) Co‐immunoprecipitation (Co‐IP) assays detecting interactions between HAP1 and the carboxy‐terminal region of IP3R1 in primary brain ECs. Cells were infected with HAP1‐HA and IP3R1‐Flag lentivirus for 4 d and harvested for Co‐IP assays. F) Western blot analysis of HA‐HAP1 expression after transfecting the HAP1‐HA together with *Scramble shRNA*, *Hap1‐shRNA1*, or *Hap1‐shRNA2* plasmids in HEK293T cells to confirm knockdown efficiency. G) Calcium transient traces in wild‐type cells (blue line), *Foxq1* cKO cells (red line), and *Foxq1* cKO cells overexpressing HAP1 (green line) triggered by m‐3M3FBS.H) Histogram depicting the average amplitudes of m‐3M3FBS triggered Ca^2+^ transients, represented by ΔF/F0 of Fluo‐4 (*n* = 11 cells from two independent experiments). I) Measurement of the NAD^+^/NADH ratios under HAP1 knockdown in primary brain ECs (*n* = 5 replications). J) Measurement of the NAD^+^/NADH in wild type, *Foxq1* cKO, and *Foxq1* cKO with overexpression of HAP1 in brain ECs (*n* = 5 replications). Data are shown as mean ± s.e.m for (F, H, I, and J). ** *P* < 0.01, *** *P* < 0.001. Two‐tailed unpaired Students’ *t*‐test was used.

Transmission electron microscopy analysis of brain endothelial cells revealed extensive close spatial proximity between ER and mitochondria (**Figure** **5**A), forming the structural foundation for inter‐organellar communication. These membrane contact sites are evolutionarily conserved platforms that facilitate rapid calcium transfer and metabolic coordination between organelles. The ER serves as the primary intracellular calcium reservoir, sequestering approximately 70% of total cellular calcium.^[^ ^24^ ^]^ Upon extracellular stimulation, phospholipase C (PLC) activation generates inositol 1,4,5‐trisphosphate (IP3), which binds to and activates IP3 receptors clustered on the ER membrane, triggering localized calcium release events (Figure 5B). This calcium mobilization system enables rapid conversion of extracellular signals into intracellular calcium transients that can directly modulate mitochondrial metabolism.

To assess calcium signaling capacity in brain endothelial cells, we employed m‐3M3FBS, a potent PLC activator, to stimulate IP3‐dependent calcium release.^[^ ^25^ ^]^ Wild‐type primary brain endothelial cells responded with robust, high‐amplitude calcium transients (Figure 5C), confirming the presence of functional calcium mobilization machinery. Co‐immunoprecipitation experiments validated the physical interaction between HAP1 and IP3R1 in brain endothelial cells (Figure 5E), establishing that the HAP1‐IP3R1 regulatory complex previously characterized in neurons is also functionally conserved in the brain vasculature. To directly assess HAP1's regulatory role, we performed siRNA‐mediated Hap1 knockdown, which resulted in significantly diminished calcium transient amplitudes (Figure 5C,D,F). This finding demonstrates that HAP1 is not merely associated with IP3R1 but actively enhances calcium release efficiency in brain endothelial cells. Consistent with our transcriptional analysis showing reduced *Hap1* expression in *Foxq1* knockout cells, calcium imaging revealed that *Foxq1* conditional knockout brain endothelial cells exhibited substantially diminished calcium transients compared to wild‐type controls (Figure 5G,H). Critically, this calcium signaling defect could be rescued by HAP1 overexpression, providing definitive evidence that FOXQ1 regulates calcium homeostasis specifically through transcriptional control of Hap1 expression.

The functional significance of ER‐mitochondrial calcium transfer extends beyond signaling to direct metabolic regulation. Mitochondrial calcium uptake is essential for optimal tricarboxylic acid (TCA) cycle function, as calcium ions serve as allosteric activators of key rate‐limiting enzymes including isocitrate dehydrogenase, alpha‐ketoglutarate dehydrogenase, and pyruvate dehydrogenase.^[^ ^26^ ^]^ To assess TCA cycle activity, we measured the NAD^+^/NADH ratio, a sensitive indicator of mitochondrial oxidative metabolism. Both *Foxq1* knockout and *Hap1* knockdown resulted in significant reductions in the NAD^+^/NADH ratio (Figure 5I,J), indicating impaired TCA cycle flux. Importantly, HAP1 overexpression in *Foxq1* knockout cells partially restored the NAD^+^/NADH ratio (Figure 5I,J), demonstrating that calcium signaling defects contribute mechanistically to the observed mitochondrial dysfunction.

Collectively, these findings reveal a sophisticated regulatory mechanism whereby FOXQ1 coordinates mitochondrial metabolic activity through transcriptional control of calcium signaling machinery. By regulating *Hap1* expression, FOXQ1 modulates the efficiency of ER‐mitochondrial calcium transfer, which in turn controls TCA cycle activity and oxidative phosphorylation capacity in brain ECs.

### 2.7 FOXQ1 Maintains Mitochondrial Ultrastructure and Respiratory Efficiency Through ADCK1‐Mediated Cristae Organization

FOXQ1 target transcripts were also implicated in the regulation of mitochondrial fusion, identifying several direct targets of FOXQ1, including *Adck1*, *Oma1*, and *Bnip3*. While OMA1 and BNIP3 are known as mitochondrial quality control proteins that respond to cellular stress, ADCK1 was reported to be essential for mitochondrial structure in *Drosophila* muscles, though its specific role in mammalian brain endothelial cells remained unexplored.^[^ ^27^ ^]^ Given the profound mitochondrial morphological abnormalities observed in *Foxq1*‐deficient cells, we hypothesized that ADCK1 might serve as a critical effector of FOXQ1‐mediated mitochondrial structural regulation.

**Figure 6:**
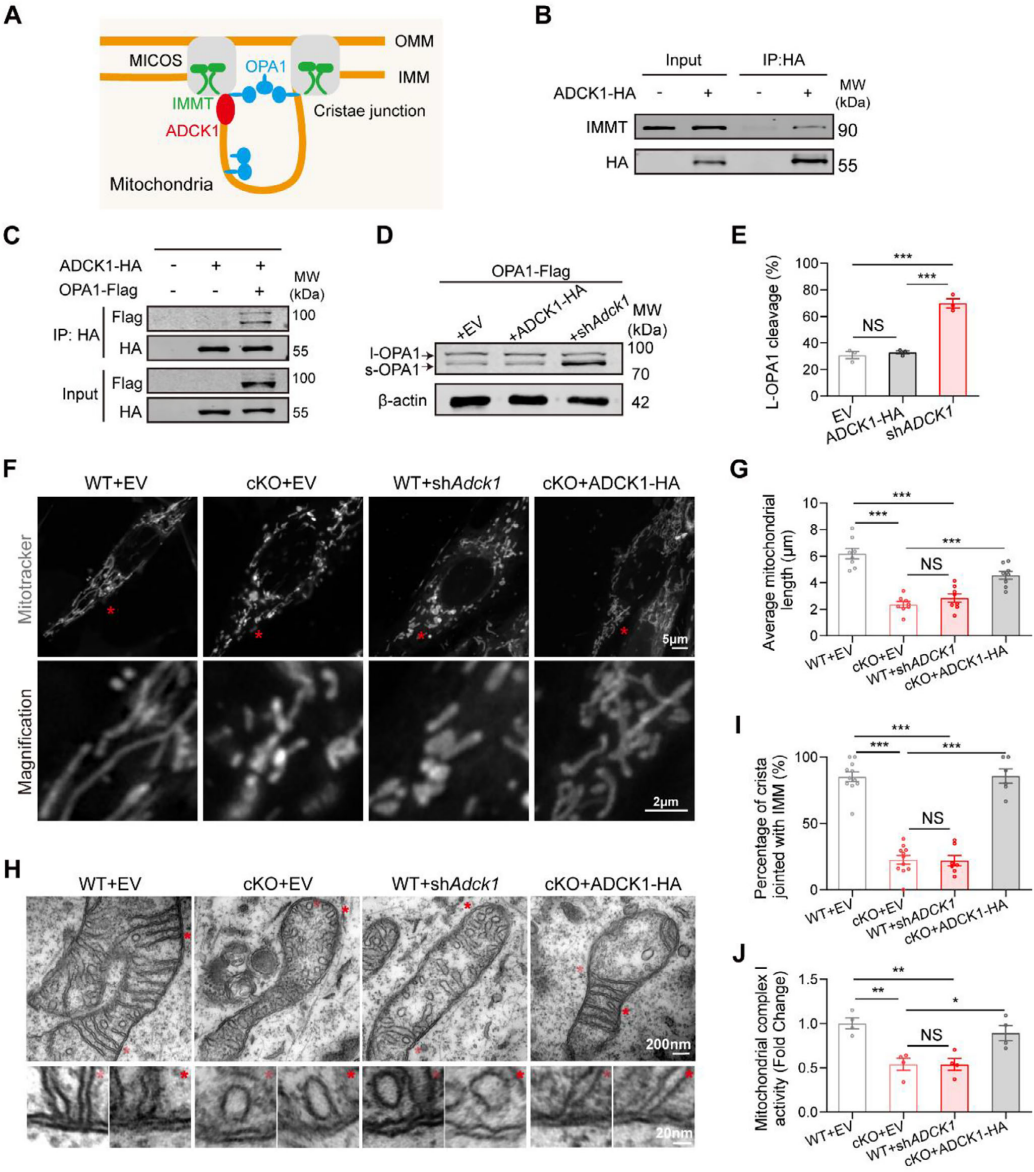
FOXQ1 maintains the mitochondrial cristae by enhancing the expression of *Adck1*. A) Schematic diagram for the structure of mitochondrial crista junction. Highlights include the role of IMMT (Mic60), a core component of the MICOS complex (Mitochondrial Contact Site and Cristae Organizing System), which regulates crista junction formation and the interface between the outer mitochondrial membrane (OMM) and inner mitochondrial membrane (IMM). OPA1 is depicted controlling crista width, with a balance of its long and short forms essential for maintaining tight crista junctions. B) Co‐IP assay detecting interaction between ADCK1 and IMMT in primary brain ECs. Cells were infected with ADCK1‐HA lentivirus for 4 d and harvested for Co‐IP assays. C) Co‐IP assay detecting interactions between ADCK1 and OPA1 in primary brain ECs. Cells were infected with ADCK1‐HA and OPA1‐Flag lentivirus for 4 d and harvested for Co‐IP assays. D) Immunoblot images showing the levels of OPA1 in primary bECs infected with OPA1‐Flag and with either the empty vector, ADCK1‐HA, or *Adck1‐shRNA* lentivirus. Arrows indicate the membrane‐anchored long isoform (L‐OPA1) and a soluble short isoform (S‐OPA1) of OPA1. E) Quantification of the percentage of the L‐OPA1 cleavage (*n* = 3 independence experiments). F) Representative images of mitotracker‐labeled mitochondria in primary ECs of wild type, *Foxq1* cKO, wild type introduced with *Adck1*‐*shRNA*, and *Foxq1* cKO introduced with ADCK1‐HA. Scale bars, 5 µm (above) and 2 µm (below). G) Quantification of average mitochondrial length. Each data point represents the average mitochondrial size per individual cell (*n* = 8 cells). H) TEM images of the mitochondria in primary brain ECs of wild type, *Foxq1* cKO, wild type introduced with *Adck1*‐*shRNA*, and *Foxq1* cKO introduced with ADCK1‐HA. Bottom row shows the magnified views of mitochondrial crista. Scale bars, 200 nm (above) and 20 nm (below). I) Quantification of the percentage of the cristae junctions connected to the inner mitochondrial membrane. Each data point represents the percentage per individual mitochondria (*n* = 11 mitochondria). Only mitochondria with crista greater than 10 were analyzed. J) Quantification of the mitochondrial complex I activity (*n* = 4 replications). Data are shown as E,G,I,J) mean ± s.e.m. *P < 0.05, **P < 0.01, ***P < 0.001. Two‐tailed unpaired Students’ *t*‐test was used.

We first confirmed that mouse ADCK1 was localized to the mitochondrial inner membrane in primary brain ECs (Figure S6A, Supporting Information). To understand ADCK1's molecular function, we interrogated the BioGRID protein interaction database and identified two key binding partners: IMMT (Inner Mitochondrial Membrane protein) and YME1L (ATP‐dependent zinc metalloprotease). IMMT serves as a core component of the mitochondrial contact site and cristae organizing system (MICOS), a multi‐protein complex that is absolutely essential for cristae formation, stabilization, and maintenance of proper inner membrane architecture (**Figure** **6**A). YME1L, conversely, functions as a critical regulatory protease that controls the proteolytic processing of OPA1 (Optic Atrophy 1), determining the balance between the long form (L‐OPA1) and short form (S‐OPA1). This OPA1 processing directly regulates cristae remodeling dynamics and mitochondrial fusion capacity, making YME1L a key determinant of mitochondrial morphological plasticity.

Co‐immunoprecipitation experiments validated the predicted protein interactions, confirming that ADCK1 forms stable complexes with both IMMT (Figure 6B) and OPA1 (Figure 6C) in brain endothelial cells. These interactions position ADCK1 as a potential coordinator between the MICOS cristae organizing system and the OPA1‐dependent fusion machinery.

The biochemical changes in OPA1 processing translated into profound ultrastructural alterations. Transmission electron microscopy revealed that *Adck1* knockdown induced mitochondrial fragmentation and conversion of the normal lamellar cristae architecture into aberrant vesicular‐like structures (Figure 6F–I). These morphological changes are characteristic of mitochondrial dysfunction and impaired respiratory capacity. Critically, *Foxq1* knockout cells exhibited an identical ultrastructural phenotype, and this could be completely rescued by lentiviral overexpression of ADCK1 (Figure 6F–I and Figure S6D, Supporting Information). These results demonstrated that FOXQ1 regulates *Adck1* expression to stabilize mitochondrial cristae structures.

Tightly packed lamellar cristae are essential for optimal spatial organization of electron transport chain (ETC) complexes and ATP synthase, creating the precise membrane topology required for efficient oxidative phosphorylation.^[^ ^28^ ^]^ The conversion to vesicular cristae fundamentally disrupts this organization, compromising respiratory complex assembly and function. To directly assess the functional consequences of cristae disorganization, we measured the enzymatic activity of mitochondrial Complex I, the rate‐limiting component of the electron transport chain.^[^ ^29^ ^]^ Both Foxq1 knockout and Adck1 knockdown resulted in significant reductions in Complex I activity (Figure 6J), providing biochemical evidence that structural alterations directly impair respiratory function. ADCK1 overexpression in Foxq1 conditional knockout cells restored Complex I activity (Figure 6J), demonstrating that the respiratory defects are mechanistically linked to cristae disorganization.

We found that the loss of either FOXQ1 or ADCK1 significantly reduced the Complex I activity (Figure 6J). Overexpression of ADCK1 in *Foxq1* cKO cells restored Complex I activity (Figure 6J). Together, these results illustrate that FOXQ1 regulates ADCK1 to stabilize mitochondrial cristae structures for efficient electron transport, which is essential for mitochondrial metabolic activity.

Together, these findings reveal that FOXQ1 preserves mitochondrial respiratory efficiency by transcriptionally regulating the cristae‐organizing machinery. Through control of *Adck1* expression, FOXQ1 ensures proper OPA1 processing, cristae structure, and the spatial organization of respiratory complexes. The FOXQ1‐ADCK1 regulatory axis thus represents a critical quality control mechanism ensuring that brain ECs maintain the mitochondrial structural integrity necessary for their high metabolic demands.

## 3 Discussion

Our study reveals FOXQ1 as a previously unrecognized transcriptional regulator that orchestrates the unique metabolic program required for brain endothelial cell function. Unlike the prevailing model that endothelial cells rely primarily on glycolysis, our findings demonstrate that brain ECs exhibit specialized mitochondrial‐dependent metabolism, with FOXQ1 serving as a critical coordinator of this metabolic adaptation. The transcriptional enrichment of mitochondrial genes in brain ECs, coupled with their exceptionally high mitochondrial volume, supports the concept that these cells have evolved distinct bioenergetic strategies to meet the extraordinary metabolic demands of the blood‐brain barrier.

The selective expression of FOXQ1 in brain ECs, but not in peripheral endothelium, suggests an evolutionary adaptation to the unique physiological requirements of the central nervous system. The brain's disproportionate consumption of oxygen (20% of total body oxygen despite representing only 2% of body mass) necessitates highly efficient vascular support systems. Our demonstration that FOXQ1 deletion leads to compromised mitochondrial function while preserving structural BBB integrity indicates that metabolic competence and barrier function are separable but interdependent processes. FOXQ1 emerges as a specialized regulator within the Wnt/β‐catenin‐driven transcriptional program.^[^ ^30^ ^]^ While *Foxf2* and *Foxc1*, clustered neighbors of *Foxq1*, regulate BBB tight junctions and pericyte recruitment,^[^ ^10^, ^11^ ^]^ FOXQ1 uniquely fine‐tunes endothelial metabolism without affecting barrier integrity. This functional divergence suggests evolutionary selection for metabolic specialization in brain ECs, possibly linked to the brain's disproportionate energy consumption. Notably, Wnt7a/β‐catenin signaling induces *Foxq1* expression during BBB maturation,^[^ ^30^ ^]^ creating a feedforward loop where metabolic competence reinforces barrier function. This contrasts with peripheral ECs, where Wnt signaling primarily drives angiogenic glycolysis,^[^ ^31^ ^]^ highlighting tissue‐specific adaptation of conserved pathways.

Our mechanistic studies reveal that FOXQ1 employs two complementary strategies to maintain mitochondrial homeostasis: calcium signaling regulation through HAP1 and structural organization through ADCK1. This dual control mechanism represents a sophisticated regulatory system that ensures both metabolic flux and organellar integrity. The FOXQ1‐HAP1‐calcium axis provides dynamic control over mitochondrial bioenergetics through modulation of TCA cycle enzyme activity, as calcium serves as an allosteric activator of key rate‐limiting enzymes.^[^ ^32^ ^]^ Our demonstration that FOXQ1 regulates ER‐mitochondrial calcium transfer through HAP1 establishes a direct link between transcriptional control and metabolic output. This mechanism mirrors neuronal calcium dynamics^[^ ^33^ ^]^ but represents a novel regulatory axis in ECs. Complementing this dynamic regulation, the FOXQ1‐ADCK1 pathway provides structural stability through cristae organization. The conversion from normal lamellar to aberrant vesicular cristae in FOXQ1‐deficient cells represents a fundamental breakdown in mitochondrial architecture that directly impairs respiratory complex function, highlighting the intimate relationship between form and function in mitochondrial biology.

Intriguingly, this metabolic regulatory role sharply contrasts with FOXQ1's well‐established function in cancer, where it is widely recognized as an epithelial‐to‐mesenchymal transition (EMT)‐promoting factor that drives tumor cell migration, proliferation, and metastasis across multiple carcinomas.^[^ ^34^ ^]^ In cancer contexts, FOXQ1 transcriptionally activates core EMT drivers such as *TWIST1* and *ZEB2*, while repressing epithelial markers including *CDH1*, leading to enhanced cellular motility and invasiveness.^[^ ^35^ ^]^ Additionally, FOXQ1 promotes tumor angiogenesis via upregulation of pro‐angiogenic factors (VEGF, ANGPT1, CCL2) and facilitates vascular mimicry, supporting neoplastic dissemination.^[^ ^35^, ^36^ ^]^ In contrast, our findings demonstrate that FOXQ1 in brain endothelial cells primarily governs cellular metabolic integrity and mitochondrial homeostasis. While FOXQ1 loss resulted in profound mitochondrial dysfunction and associated cellular structural changes, including cristae disorganization and microvilli formation, it did not disrupt overall vascular architecture, angiogenic capacity, or blood‐brain barrier integrity. This suggests that FOXQ1's essential role in quiescent brain endothelium centers on maintaining cellular bioenergetic function rather than driving the dynamic morphological changes characteristic of cancer progression. These findings highlight the context‐dependent nature of FOXQ1 function: a critical metabolic regulator in the stable brain vasculature versus a pro‐migratory transcriptional driver in the dynamic cancer environment. The divergent cellular outcomes likely reflect the distinct physiological demands and regulatory networks operating in quiescent endothelium compared to the highly proliferative and invasive cancer microenvironment. Importantly, our behavioral data suggest that even subtle metabolic dysfunction in brain endothelial cells can have profound consequences for neurological function, emphasizing the critical importance of maintaining optimal endothelial cell metabolism for brain health.

Despite preserved blood‐brain barrier integrity and normal vascular architecture, these mice exhibited motor coordination deficits, impaired spatial memory, and altered exploratory behavior. This disconnect suggests that endothelial metabolic dysfunction can manifest as neurological symptoms through mechanisms independent of overt barrier breakdown, potentially involving altered neurovascular coupling or compromised endothelial support for neuronal metabolism. This finding has important implications for understanding the pathogenesis of cerebrovascular diseases, where metabolic dysfunction often precedes structural vascular changes. Our findings may be particularly relevant to age‐related neurodegenerative diseases, where cerebrovascular dysfunction and metabolic impairment are increasingly recognized as early pathological events.^[^ ^37^ ^]^ The decline in mitochondrial function with aging could render brain ECs particularly vulnerable to further metabolic stress, potentially creating a cascade of vascular dysfunction that contributes to cognitive decline.

While our study provides compelling evidence for FOXQ1's role in brain EC metabolism, several limitations should be acknowledged. The conditional knockout approach, while tissue‐specific, eliminates FOXQ1 throughout development, potentially masking adaptive responses. Future studies using inducible knockout systems could better distinguish acute versus developmental roles of FOXQ1. Additionally, our focus on mitochondrial function, while mechanistically important, does not fully capture the complexity of brain EC physiology. Future investigations should examine how FOXQ1‐mediated metabolic control integrates with other critical EC functions, including inflammatory responses, angiogenesis, and neurovascular coupling. The translational relevance of our findings would be strengthened by validation in human brain EC models and examination of FOXQ1 expression patterns in human cerebrovascular disease. Development of FOXQ1‐specific biomarkers could facilitate clinical studies and provide tools for monitoring therapeutic interventions targeting this pathway.

## 4 Conclusion

Our study establishes FOXQ1 as a critical transcriptional coordinator of the specialized metabolic program that distinguishes brain endothelial cells from their peripheral counterparts. Through coordinated regulation of calcium signaling and mitochondrial structural organization, FOXQ1 ensures that brain ECs maintain the bioenergetic capacity necessary for blood‐brain barrier function. These findings not only advance our fundamental understanding of cerebrovascular biology but also identify novel potential therapeutic targets for treating neurological diseases characterized by vascular dysfunction. The FOXQ1 regulatory network represents a promising paradigm for developing metabolism‐based interventions that may preserve or restore brain endothelial function in disease states.

## 5 Experimental Section

### Mouse

The *Foxq1* conditional knockout mice were generated via the CRISPR‐CAS9‐mediated double nicking strategy. The guide RNA sequences were as follows: gRNA1 (5′‐*ACGCAGCGCGCAGCTCAGGACGG*‐3′) and gRNA2 (5′‐*GCCTGTACTCTCCCGCATAGAGG*‐3′). Tie2‐cre mice were employed to recognize and cleave the flox sites. Littermates lacking Cre transgene served as controls. Both male and female mice aged 8–16 weeks were used in experiments. All animal experiments were approved by the Animal Care and Use Committees of the Institute of Zoology, Chinese Academy of Sciences. The ethical approval number for animal studies is IOZ‐IACUC‐2024‐143.

### Plasmids

Mouse *Foxq1* cDNA (accession number NM_008239), mouse *Hap1* cDNA (accession number NM_010404), mouse *Adck1* cDNA (accession number NM_001277296), and mouse *Opa1* cDNA (accession number NM_001199177) were obtained by polymerase chain reaction (PCR) and cloned into *pCDH‐CMV* to generate *PCDH‐CMV‐HA‐FOXQ1*, *PCDH‐CMV‐HAP1‐HA*, *PCDH‐CMV‐ADCK1‐HA*, and *PCDH‐CMV‐OPA1‐Flag*. The DNA fragment of mouse *TOM20* was prepared by PCR and constructed into *pCDH‐LC3B‐mCherry‐GFP* (MiaoLingPlasmid, China # P50847) to replace *LC3B* in generating the *pCDH‐TOM20‐mCherry‐GFP*. The DNA fragment of the carboxy‐terminal region (124 amino acids F2626‐A2749) of mouse *Ip3r1* (accession number NM_010585) was obtained by PCR and cloned into *pCDH‐CMV* to generate *PCDH‐CMV‐IP3R‐Flag*. The shRNA fragments were synthesized and constructed into *pSicoR*. The sequences of shRNAs were follows: *Foxq1*, 5′‐ *GGACAACTACTGGATGCTCAA*‐3′; *Adck1*, 5′‐*GACTCAGAGATTCGCAATAAT*‐3′; *Hap1*, 5′‐*CCCACGAAGAAGATCACCGAA*‐3′ and 5′‐*AGGAGAATAATAAGCTGGAAA*‐3′.

For lentiviral production, target plasmids were co‐transfected with lentiviral packaging plasmids, *pMD2.G* (envelope plasmid) and *psPAX2* (packaging plasmid), into 293T cells. After transfection, the supernatant containing lentiviral particles was harvested and used to infect primary brain ECs. To enhance transduction efficiency, 8 µg mL^−1^ of polybrene was included during the infection.

### Immunostaining

Brains were fixed in 4% paraformaldehyde (PFA) in phosphate buffered saline (PBS) overnight and subsequently transferred to 30% sucrose for dehydration. The brains were then sectioned at a thickness of 40 µm for immunostaining. For immunostaining, brain slices or cell samples were fixed in 4% PFA at room temperature for 20 min, followed by three washes with 0.1% PBST (PBS containing 0.1% Triton X‐100). The samples were blocked with 5% bovine serum albumin (BSA) in 0.1% PBST for 1 h at room temperature, then incubated with primary antibodies at 4 °C overnight. Following three 10 min washes in PBS, the samples were incubated with secondary antibodies for 2 h at room temperature, and subsequently stained with DAPI (Sigma, Cat# D9542) for 2 min to label nuclei. Finally, the slices were mounted using 50% glycerol.

### Antibodies

The following antibodies were used for immunostaining: biotinylated anti‐IB4 (Vector Laboratories; Cat# B‐1205; RRID: AB_2314661), mouse anti‐FOXQ1 (Santa Cruz Biotechnology; Cat# sc‐166265; RRID: AB_2105178), rabbit anti‐TOM20 (Beyotime, Cat# AF1717), rabbit anti‐Collagen IV (Bioss; Cat# BS‐4595R; RRID: AB_11101792), rabbit anti‐BLBP (Abcam; Cat# ab32423, RRID: AB_880078), rat anti‐PDGFRß (BD Bioscience; Cat# 558774; RRID: AB_397117), rat anti‐LAMP1 (BD Biosciences; Cat# 553792; RRID: AB_2134499), Rabbit anti‐Ki67 (Abcam; Cat# ab15580; RRID: AB_443209), and rabbit anti‐HA (Cell Signaling Technology; Cat# 3724S; RRID: AB_1549585). The following antibodies were used for western blotting and Co‐IP experiment: rabbit anti‐HA (Cell Signaling Technology; Cat# 3724S; RRID: AB_1549585), rabbit anti‐β‐actin (Proteintech; Cat# 20536‐1‐AP; RRID: AB_10700003), mouse anti‐Flag (Sigma‐Aldrich; Cat# F1804; RRID: AB_ 262044), and mouse anti‐OPA1 (Proteintech; Cat# 10179‐1‐AP; RRID: AB_2127193). The following antibodies were used for CUT&Tag experiment: rabbit anti‐HA (Cell Signaling Technology; Cat# 3724S; RRID: AB_1549585), and rabbit anti‐H3K4me3 (Active Motif; Cat# 39160; RRID: AB_ 2615077).

### Primary Mouse Brain Endothelial Cell Culture

Primary mouse brain ECs were isolated as previously described.^[^ ^38^ ^]^ Briefly, E18 brains was dissected under stereomicroscopy to harvest the cerebral cortex. This tissue was then digested with 20 U per mg papain (Worthington, Cat#Ls003119) for 5 min at 37 °C. The resulting mixture was strained through a 70 µm nylon mesh. Cells were then exposed to 2 mL of red blood cell lysing buffer (Sigma‐Aldrich) for 2 min to remove erythrocytes. The cell suspension were then incubated with anti‐CD31‐FITC (Biolegend, Cat#102406) at 4 °C for 30 min. These labeled cells were subsequently sorted using a FACSCalibur cytometer (Becton Dickinson). The isolated primary endothelial cells were then cultured on plates coated with type I collagen (Sigma‐Aldrich) within ECM media (Sciencell, Cat#1001). Fresh medium was supplied to the cultures every two to 3 d.

### Transmission Electron Microscopy (TEM) Analysis

TEM was performed according to the method previously described.^[^ ^39^ ^]^ Briefly, wild‐type and *Foxq1* ^−/−^ brains tissues were sectioned into small cubes and fixed overnight at 4 °C using electron microscopy‐grade 2% paraformaldehyde (PFA) and 2.5% glutaraldehyde in phosphate‐buffered saline (PBS, pH 7.4). For primary brain endothelial cells, samples were cultured on plastic slides and infected with lentivirus until they reached approximately 70% confluence before fixation. Following fixation, all samples were washed three times with PBS and post‐fixed in 1% osmium tetroxide for 1 h. After another series of thorough washes in PBS, the samples underwent a dehydration process through a graded ethanol series, including concentrations of 30%, 50%, 70%, 80%, 90%, 95%, and 100%. Subsequently, the samples were washed twice in 100% acetone. The samples were then soaked in a 1:1 ratio of acetone to epoxy resin for 1 h, followed by a 1:3 ratio of acetone to epoxy resin for 3 h, and finally in pure epoxy resin for more than 5 h. Finally, samples were embedded in epoxy resin and polymerized at 60 °C for 48 h. Ultra‐thin sections (60 nm) were obtained using a Leica UC7 ultramicrotome. Sections were stained with uranyl acetate and Reynold's lead citrate for contrast enhancement. The prepared sections were examined and imaged using a Tecnai G2 F20 TWIN TMP transmission electron microscope.

### Wound Healing Assay

Primary mouse brain ECs were seeded in 6‐well plates and grown to full confluence. Cells were then starved for 10 h in ECM containing 1% FBS. A sterile 200 µL pipette tip was used to create a linear scratch in the monolayer. After washing twice with DPBS to remove cell debris, cells were incubated in fresh medium. Images were captured at 0, 12 and 24 h using a phase‐contrast microscope. The wound area was quantified using ImageJ software, and the relative wound closure was calculated as (*W*_T0_ − *W*_Tx_)/*W*_T0_ × 100%, where *W*_T0_ is the wound width at time zero and *W*_Tx_ is the wound width at 12 h or 24 h after scratching.

### EdU Proliferation Assay

ECs were seeded onto coverslips in a 24‐well plate. After 24 h, cells were incubated with 10 µm 5‐ethynyl‐2′‐deoxyuridine (EdU) for 4 h. Cells were then fixed, permeabilized, and stained using the BeyoClick EdU Cell Proliferation Kit with Alexa Fluor 488 (Beyotime, China; Cat# C0071S) and Ki67 antibody according to the manufacturer's protocol. Nuclei were counterstained with DAPI. Images were captured with a confocal microscope, and the percentage of EdU‐positive cells was determined from at least five random fields per condition.

### Tube Formation Assay

A 24‐well plate was coated with 200 µL of Matrigel (Corning, #354227) and allowed to polymerize at 37 °C for 30 min. Then, 250 µL of the primary brain ECs (1.5×10^5^ cells) were seeded onto the Matrigel and incubated in a 37 °C incubator overnight. Tube formation was imaged using an inverted phase‐contrast microscope. The total tube length and number of branch points were quantified using the ImageJ software.

### Rotarod Test

All behavioral tests were conducted during the light phase (09:00–17:00) under controlled environmental conditions (22 ± 2 °C). Mice were habituated to the testing room for 30 min before experiments. The Rotarod test was conducted to assess the motor coordination and balance of the subjects. A total of 12 wild‐type and 13 *Foxq1* cKO mice, aged 8–16 weeks, were included in this study. The test was performed using a standard Rotarod apparatus (Model XR‐6C, Xinruan Co., Shanghai, China), which consists of a rotating rod with a width of 60 mm and a diameter of 30 mm. Prior to testing, the animals underwent a 3 d training period consisting of three sessions per day. The training protocol involved gradually increasing the rod's speed, starting at 4 rpm for 1 min, followed by 10 rpm for 2 min, and finally reaching 30 rpm for 3 min. If a mouse fell from the rod during training, it was given a rest period of at least 15 min to minimize stress and fatigue before continuing. In the test session, mice were placed on the rotating rod, which was set to accelerate from 4 to 40 rpm over a 5 min period. A total of three trials were conducted for each mouse, and the mean latency to fall from the rod was recorded and used for analysis.

### Morris Water Maze

The Morris Water Maze (MWM) task was used to assess spatial learning and memory in mice. The maze consists of a circular pool filled with water and made opaque with non‐toxic white paint (titanium dioxide). A hidden platform (10 cm in diameter) was submerged 1 cm below the water surface and placed in southeast (SE) quadrant. For the training phase, mice underwent 5 consecutive days of platform training, with each day consisting of four trials. In each trial, the mouse (aged 8–16 weeks) was placed in the pool at one of four starting positions, and the time to locate and climb onto the hidden platform was recorded. A total of 17 wild‐type and 15 *Foxq1* cKO mice were tested. If the mouse failed to find the platform within 60 s, it was guided to the platform and allowed to remain there for 10 s before being removed from the maze. The inter‐trial interval was set to 15–20 min. The position of the platform remained constant throughout the 5 d of training. On the 6th day, a probe trial was conducted to assess memory retention. During the probe trial, the platform was removed from the maze, and the mouse was allowed to swim freely for 60 s. The behavioral data were collected and analyzed using Panlab SMART 3.0 video tracking software.

### Open Field Test

An open field test was conducted in a square arena measuring 50 cm × 50 cm. All tests were performed under controlled lighting conditions and in a low‐noise environment to minimize external disturbances. Prior to each trial, the arena was thoroughly cleaned to eliminate any scent markers or residues from previous subjects. Rodent movements during the test were recorded using a camera system. Mice (aged 8–16 weeks) were individually placed in the center of the arena. A total of 16 wild‐type and 17 *Foxq1* cKO mice were tested. Following a 3 s habituation period, their behavior was recorded for a duration of 5 min. Automated analysis of the recorded behavior was performed using Panlab SMART 3.0 video tracking software. The central zone, defined as the area within 25 cm × 25 cm at the center of the arena, was specifically analyzed.

### TMRE Imaging and Analysis

For assessing mitochondrial membrane potential, mouse brain endothelial cells were obtained from primary culture mouse cerebral cortex as described in the previous section and plated on collagen type I (Sigma, Cat#C3867‐1VL) coated coverslip bottom dishes with grid (NEST, Cat#801002) using ECM medium (ScienCell, Cat#1001). For FOXQ1 knockdown, pSicoR‐U6 promoter‐*Foxq1* shRNA‐EF1α promoter‐GFP lentivirus with low titer were added and infected for 3 d. The cells were cultured to approximately 60% confluency and then incubated with tetramethylrhodamine ethyl ester (TMRE) (Beyotime, Cat# C2001S) at 37 °C for 45 min. Following incubation, the cells were washed twice with warm PBS to remove excess dye, and 2 mL of fresh medium was added. Fluorescence images were acquired using a Zeiss LSM 880 with Airyscan microsystem (excitation: 549 nm, emission: 574 nm). For analysis, a z summation of the stack was used to measure the whole cell TMRE signal. The background signal was subtracted using ImageJ software.

### Evans Blue BBB Leakage Test

To assess blood‐brain barrier permeability, 200 µL of Evans blue staining (2%; Aicon Biotech, Cat# A28136) was intraperitoneally injected into wild‐type and *Foxq1^−/−^* adult mice. After 24 h of circulation, the mice were anesthetized and perfused with 4% PFA. The brains were carefully dissected and imaged. The integrity of the blood‐brain barrier was evaluated by examining the tissue for the presence or absence of blue dye leakage.

### Mito‐Tracker Labeling and Mitochondrial Morphology Analysis

Mitochondria in primary brain endothelial cells (ECs) were labeled using Mito‐Tracker Deep Red FM (Beyotime; Cat# C1032). Wild‐type and *Foxq1* ^−/−^ cells were initially infected with either PCDH, PCDH‐CMV‐ADCK1‐HA, or pSicoR‐shAdck1 lentivirus. Subsequently, a 100 nm working solution of Mito‐Tracker was added to the ECM medium and incubated at 37 °C for 15 min. After incubation, the cells were washed twice with DPBS and transferred to fresh culture medium. Cells exhibiting GFP fluorescence were identified as successfully infected by the lentivirus. Imaging was conducted using a confocal microscope in Airyscan mode with a 63x oil immersion objective and a zoom factor of 2.5. The maximum length of the mitochondrial fragments was manually traced using the freehand tool in Fiji software. Only those mitochondrial fragments that could be distinctly recognized as single mitochondria were measured.

### The Oxygen Consumption Rate (OCR) and Extracellular Acidification Rate (ECAR) Measurement

The oxygen consumption rate (OCR) and extracellular acidification rate (ECAR) were measured using Seahorse XF Pro Analyzer (Agilent XFe96). Primary brain endothelial cells were plated at a density of 10000 cells per well in 80 µL growth media. OCR and ECAR were measured using Seahorse XF Cell Mito Stress Test Kit (Agilent, Cat#103015‐100) and Seahorse XF Glycolysis Stress Test Kit (Agilent, Cat#103020‐100). OCR was measured in XF medium containing 1 mm sodium pyruvate, 2 mm glutamine and 10 mm glucose under basal conditions and in response to oligomycin (1.5 µm), FCCP (0.5 µm) and rotenone/antimycin A (0.5 µm). ECAR was measured in XF medium containing 2 mm glutamine under basal conditions and in response to glucose (10 mm), oligomycin (1 µm) and 2‐deoxy‐D‐glucose (2‐DG) (50 mm). The fractions of ATP that are produced from mitochondrial oxidative phosphorylation and glycolysis were measured using Seahorse XF Real‐Time ATP Rate Assay Kit (Agilent, Cat#103592‐100).

### TUNEL Labeling

Apoptosis in brain tissue sections was detected using the TransDetect in situ Fluorescein TUNEL Apoptosis Detection Kit (Transgen; Cat# FA201). A mixture of 200 µL of labeling solution and 8 µL of TdT enzyme was incubated at 37 °C for 1 h in darkness. Following incubation, sections were washed with 1% PBST. The samples were then mounted and examined for apoptosis using a confocal microscope, employing an excitation wavelength of 450–500 nm.

### Single‐Cell Sequencing Analysis

Single‐cell sequencing data were obtained from the publicly available dataset (ArrayExpress: E‐MTAB‐8077),^[^ ^6c^ ^]^ and processed using the Seurat R package. Doublet cells were removed using the doubletFinder_v3 function from the DoubletFinder package (v.2.0.3). The data were filtered with the following criteria: nFeature_RNA>1000, nCount_RNA<30000, percent.mt<10%, percent.redcell<10%, and percent.ribo<40%. Genes with expression levels below 3, along with mitochondrial, sex‐related, red blood cell genes, and Malat1, were excluded. Data normalization was performed using the NormalizeData function with a scale factor of 10000, and variable features were identified with the FindVariableFeatures function. The data were standardized, and the effects of mitochondrial and ribosomal genes were regressed out using the ScaleData function.

### Cleavage under Targets and Tagmentation (CUT&Tag) Assay

The CUT&Tag assay was conducted with slight modifications from the original description, using a commercial kit from Vazyme (Cat#TD903). Approximately 1×10^5^ cells were initially washed with 500 µL wash buffer, followed by centrifugation at 600 *g* for 5 min. The resultant cell pellets were resuspended in 100 µL of wash buffer and incubated with 10 µL of concanavalin A‐coated magnetic beads for 10–15 min. These bead‐bound cells were then resuspended in 50 µL of antibody buffer containing either 2 µg of rabbit anti‐HA or 2 µg of rabbit anti‐H3K4me3 antibodies, followed by incubation at room temperature for 2 h or overnight at 4 °C. After secondary antibody incubation, the cells underwent three washes with 200 µL of Dig‐wash buffer and were incubated with 2 µL of pA/G‐Tnp and 98 µL of Dig‐300 buffer for 1 h. Subsequent washes involved three cycles with 200 µL of Dig‐300 buffer before adding 10 µL of 5× TTBL mixed with 40 µL of Dig‐300 buffer and incubating at 37 °C for 1 h. The reactions were quenched using 5 µl of 20 mg mL^−1^ Proteinase K, 100 µL Buffer L/B, and 20 µL DNA extraction beads at 55 °C for 10 min. The beads were washed once with 200 µL Buffer WA, twice with 200 µL Buffer WB, and finally resuspended in 22 µL of nuclease‐free water. For library amplification, 15 µL of the purified DNA was combined with 25 µL of 2×CAM and 5 µL of barcoded primers, then amplified using a thermal cycler. PCR products were purified with VAHTS DNA Clean Beads and eluted in 22 µL of ddH_2_O. Libraries were sequenced on the Illumina NovaSeq 6000 platform.

### CUT&Tag Data Analysis

The following antibodies were used for CUT&Tag experiment: rabbit anti‐HA (Cell Signaling Technology; Cat# 3724S; RRID: AB_1549585), and rabbit anti‐H3K4me3 (Active Motif; Cat# 39160; RRID: AB_ 2615077). Raw CUT&Tag sequencing data were processed for quality control and adapter trimming using fastp (v.0.20). Paired‐end reads (R1 and R2) were screened for potential contaminants with FastQ Screen (v.0.15.2), and trimmed reads were aligned to the mouse reference genome (mm10) using Bowtie2 (v.2.4.1). Aligned BAM files were sorted and duplicates marked using Sambamba (v.0.8.2). Peak calling was executed using GoPeaks (v.1.0.0), and coverage tracks in BigWig format were generated via the bamCoverage function of deepTools (v.3.5.1). Heat maps were created using computeMatrix and plotHeatmap functions from deepTools (v.3.5.1). Peaks were annotated using ChIPseeker (v.1.30.3), referencing the TxDb.Mmusculus.UCSC.mm10.knownGene (v.3.10.0) and org.Mm.eg.db (v.3.14.0) gene annotation databases. Peaks within 3000 bp of transcription start sites (TSS) were annotated with annotatePeak, and intersections of H3K4me3 and FOXQ1 peaks were analyzed with bedtools (v.2.30.0) intersect command, followed by grouping, merging, and filtering to identify promoter region peaks. Venn diagrams illustrating the overlap between H3K4me3 and FOXQ1 genes were created using the VennDiagram package (v.1.7.3). GO enrichment analysis was conducted using clusterProfiler (v.4.2.2) with the enrichGO function to analyze the intersecting genes of H3K4me3 and FOXQ1 for GO biological process (BP) enrichment. Data deposited at GEO accession: GSE271829.

### Alphafold3 Prediction of FOXQ1–DNA Interaction

The amino acid sequence of the mouse FOXQ1 transcription factor was obtained from the Universal Protein Knowledgebase (RefSeq accession number: NP_032265.3). DNA sequences from the promoters of *Adck1* and *Hap1*, known to interact with FOXQ1, were identified using CUT&Tag analysis. The tertiary structure of FOXQ1 was predicted using AlphaFold3, an AI‐driven protein structure prediction tool developed by DeepMind.^[^ ^22^ ^]^ This structure prediction was accessed via http://alphafoldserver.com. Utilizing the predicted protein structure, docking simulations were conducted to model interactions with the target DNA sequences. These simulations considered factors such as electrostatic potential and molecular complementarity. Visualization of the results was achieved using the VScode software, equipped with a Protein Viewer plugin, which helped in identifying crucial binding motifs and interaction hotspots between FOXQ1 and the DNA.

### RNA Sequencing

The cell samples were isolated from E18 wild‐type and *Foxq1* cKO cortices using a FACSCalibur Flow Cytometer (Becton Dickinson) with CD31‐FITC antibody (Biolegend, 102406). RNA samples were extracted with TRIzol reagent, and the quality of RNA‐seq libraries was evaluated with Agilent 2100 Bioanalyzer. Sequencing was performed on an Illumina platform by Annoroad Gene Tech. Co. Ltd. (Beijing, China). Data deposited at GEO accession: GSE271827. GO analysis was performed using Metascape (http://metascape.org).

### Quantitative Real‐Time PCR

RNA was extracted from wild‐type and *Foxq1* ^−/−^ primary brain endothelial cells using TRIzol reagent. The purified RNA was reverse‐transcribed into cDNA using the First‐Strand cDNA Synthesis Kit (Tiangen, Cat# KR106‐02). Quantitative RT‐PCR was performed with the SuperReal PreMix Plus (SYBR Green) Kit (Tiangen, Cat# FP205‐03) on a RT‐PCR Detection System (LI‐COR Biosciences). GAPDH served as the internal control for normalizing mRNA levels. All primer sequences are listed in Table S2 (Supporting Information).

### Ca^2+^ Measurement

Cells were incubated with 4 µm Fluo‐4‐AM at 37 °C for 40 min. Following incubation, the medium containing Fluo‐4‐AM was removed, and the cells were washed three times with DPBS, followed by a 10 min de‐esterification period. To evoke a calcium transient, 2,4,6‐trimethyl‐N‐(meta‐3‐trifluoromethylphenyl)benzenesulfonamide (m‐3M3FBS, 20 µm) was added to the medium. Fluo‐4 was excited at 488 nm, and emission was collected at 512–520 nm using a Zeiss LSM 880 with Airyscan microsystem. Images were captured at 2 s intervals and analyzed using ImageJ software (NIH, USA). The change in cytosolic calcium concentration was quantified using Δ*F*/*F*0, with data presented as mean ± s.e.m.

### Measurement of Whole‐Cellular NAD^+^/NADH

To measure whole cellular NAD/NADH levels, the Enhanced NAD^+^/NADH Assay Kit with WST‐8 (Beyotime, Cat#S0176S) was used and optimized for endothelial cells. Wild‐type and *Foxq1* ^−/−^ cells were cultured on collagen type I (Sigma, Cat#C3867‐1VL) coated 6 well plates as described above. For HAP1 knockdown or overexpression, cells were infected with *pSicoR‐U6 promoter‐*sh*HAP1* and *pCDH‐CMV promoter‐HAP1‐HA* lentivirus for 3 d. The cells were cultured to 80% confluency, and NAD/NADH levels were determined according to the manufacturer's instructions. Cells were lysed with 200 µL of ice‐cold lysis buffer and centrifuged at 12000 *g* for 10 min at 4 °C. The supernatant was collected as the sample. To measure NADH levels, the lysate was diluted two‐fold, and 20 µL sample was added to a 96‐well plate, with five replications. To measure total NAD^+^/NADH, the lysed cell suspension was incubated at 60 °C for 30 min, then diluted twofold, and 20 µL sample was added to a 96‐well plate, with five replications. Subsequently, 90 µL of alcohol dehydrogenase was added and incubated at 37 °C for 10 mi. After that, 10 µL of color reagent was added and incubated at 37 °C for 30 min. The absorbance values were measured at 450 nm, and the levels were calculated using the generated standard curve. The levels of NAD^+^ were derived by subtracting the NADH level from the total NAD^+^/NADH level.

### Mitochondrial Complex I Activity Assay

The ratio of mitochondrial complex I activity was measured using a Mitochondrial Complex I Activity Assay kit (Bioss, Cat# AK205) according to the instructions of the manufacturer. Cells were homogenized in 1 mL extraction buffer on ice and centrifuged at 600 g for 10 min at 4 °C. The supernatant was further centrifuged at 11 000 *g* for 15 min to pellet mitochondria. The mitochondrial pellet was resuspended in 400 µL extraction buffer and sonicated for enzyme activity and protein quantification. For the activity assay, 10 µL of sample was mixed with 154 µL of AK205‐A, 20 µL of working solution, and 16 µL of AK205‐D in a UV 96‐well plate. Absorbance at 340 nm was recorded at 10 s (A1) and after a 2 min incubation at 37 °C (A2), and ΔA (A1–A2) was calculated to assess complex I activity.

### Western Blot

Sample tissues or cells were lysed in RIPA lysis buffer supplemented with 1% protease inhibitor cocktail and 1% PMSF. The lysates were sonicated 4–5 times for 10 s each, then centrifuged at 8000 rpm for 10 min. The supernatant was aspirated, and 4× loading buffer was added to the lysate, followed by boiling for 10 min. The samples were then loaded onto 10% or 12% SDS‐PAGE gels for electrophoresis. Following electrophoresis, proteins were transferred onto PVDF membranes. The membranes were blocked with 5% milk in PBST (PBS with 0.05% Tween‐20) for 1 h at room temperature. The membranes were then incubated with primary antibodies at 4 °C overnight. The next day, the membranes were washed three times with 0.05% PBST and incubated with secondary antibodies at room temperature for 1 h. Finally, the membranes were washed three times with 0.05% PBST. Protein bands were visualized and analyzed using the Odyssey imaging system (LI‐COR).

### Co‐Immunoprecipitation (Co‐IP)

For Co‐IP experiments, primary brain ECs were harvested 48 h post‐lentivirus infection. Cells were resuspended in 150 µL of IP lysis buffer and subjected to sonication 4–5 times for 10 s each, with cooling intervals on ice. A 20 µL aliquot of the lysate was reserved as the input sample and subsequently boiled. HA‐beads were added to the remainder of the lysate and incubated at 4 °C overnight. The following day, the supernatant was discarded, and the beads were retained for immunoprecipitation. These beads were washed with PBS, resuspended in 150 µL of RIPA buffer, and boiled for 10 min. The immunoprecipitated proteins were then analyzed via Western blotting to examine protein interactions.

### Statistical Analysis

Images were processed and analyzed using ImageJ software (NIH, USA). Statistical analyses were conducted using GraphPad Prism 8. All results were presented as mean ± SEM. Details regarding parameters and the number of repetitions were provided in the figure legends. Statistical comparisons between two groups were performed using an unpaired two‐tailed Student's *t*‐test. For multiple comparisons, data were analyzed using either one‐way or two‐way ANOVA. Significance levels were denoted as follows: * *P* < 0.05, ** *P* < 0.01, *** *P* < 0.001; NS indicates no significant difference.

## Conflict of Interest

The authors declare no conflict of interest.

## Author Contributions

W.Z., Y.L., L.L., and S.Z. contributed equally to this work. W.Z. and Y.L. conceived the project; W.Z., Y.L., L.L., and J.L. performed the experiments; W.Z. and S.Z. analyzed the high‐throughput sequencing data; C.W. and E.H. performed BCAS experiments; J.J. and J.Z. supervised the project; J.J., J.Z., and W.Z. acquired the funding support. W.Z. and Y.L. wrote this manuscript with input from all authors.

## Supporting information



Supporting Information

## Data Availability

The data that support the findings of this study are available from the corresponding author upon reasonable request.
