# Supplementary material for: FOXQ1 Regulates Brain Endothelial Mitochondrial Function by Orchestrating Calcium Signaling and Cristae Morphology
Source: Adv Sci (Weinh). 2025 Aug 30;12(42):e03082. doi: 10.1002/advs.202503082 (PMC12622422; doi:10.1002/advs.202503082)
Supplement: Supplementary file 1 — Supporting Information [file ADVS-12-e03082-s001.docx]

**Supplementary Information**

**FOXQ1 Regulates Brain Endothelial Mitochondrial Function by Orchestrating Calcium Signaling and Cristae Morphology**

Wenzheng Zou et al.

Corresponding authors: Jingjing Zhang, [jingjing.zhang@live.com](mailto:jingjing.zhang@live.com); Jianwei Jiao, [jwjiao@ioz.ac.cn](mailto:jwjiao@ioz.ac.cn)

This file includes:

Supplementary Figure 1-6

Supplementary Table 1-2


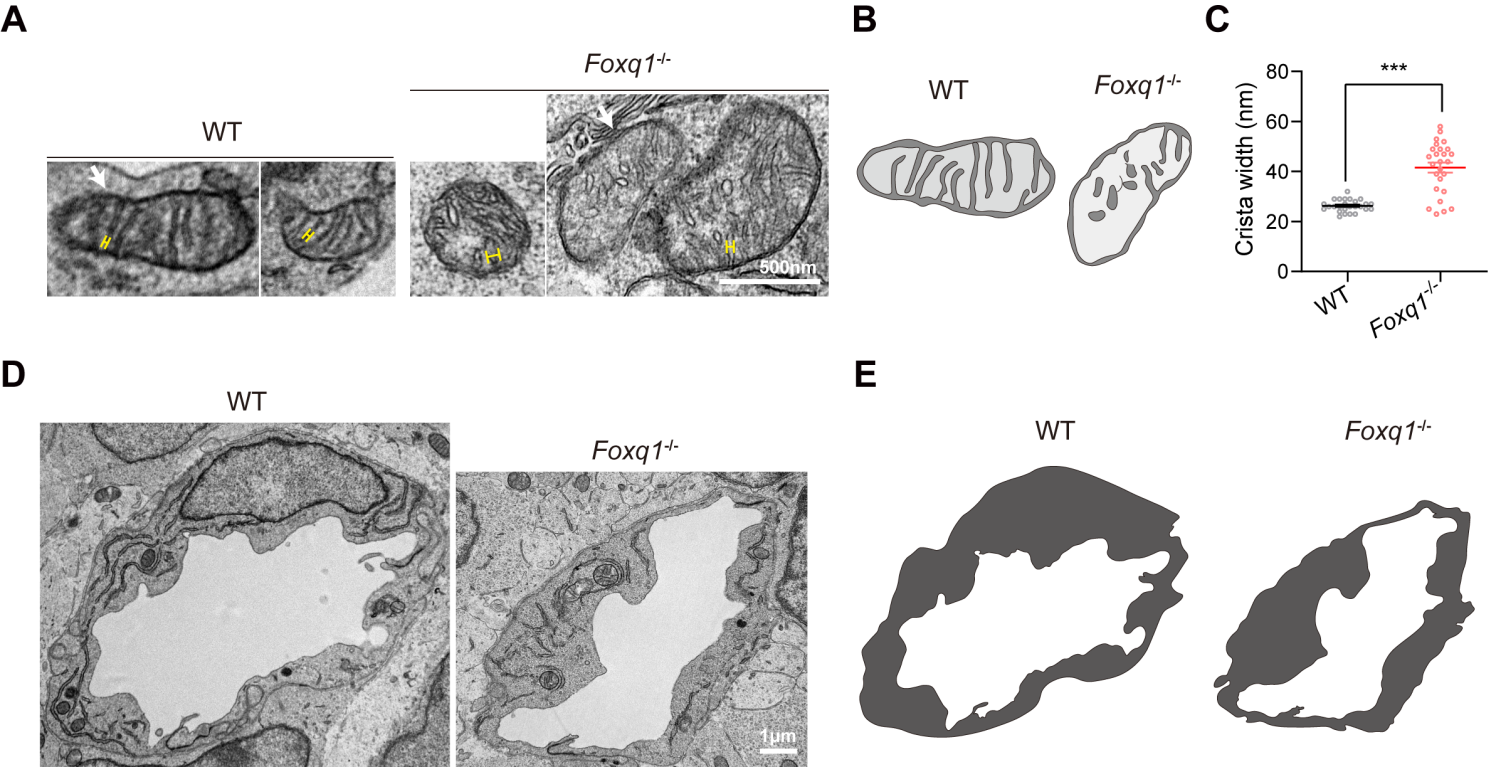


**Supplementary Figure 1.** Mitochondrial defect severity correlates with ultrastructural abnormalities in FOXQ1-deficient brain ECs. A,B) Additional TEM images and schematic diagram of mitochondria in the E17 wild-type and *Foxq1* cKO brain blood vessel. Note the increased crista width in *Foxq1* cKO brain blood vessel. Scale bar, 500 nm. C) Quantification of mitochondrial crista width (*n* = 26 crista per group from two independent experiments). D,E) Additional TEM images and schematic diagram of the E17 wild-type and *Foxq1* cKO brain blood vessels. Scale bar, 1 µm. Data are shown as mean ± s.e.m. ****P* < 0.001. Two-tailed unpaired student’s *t*-test.


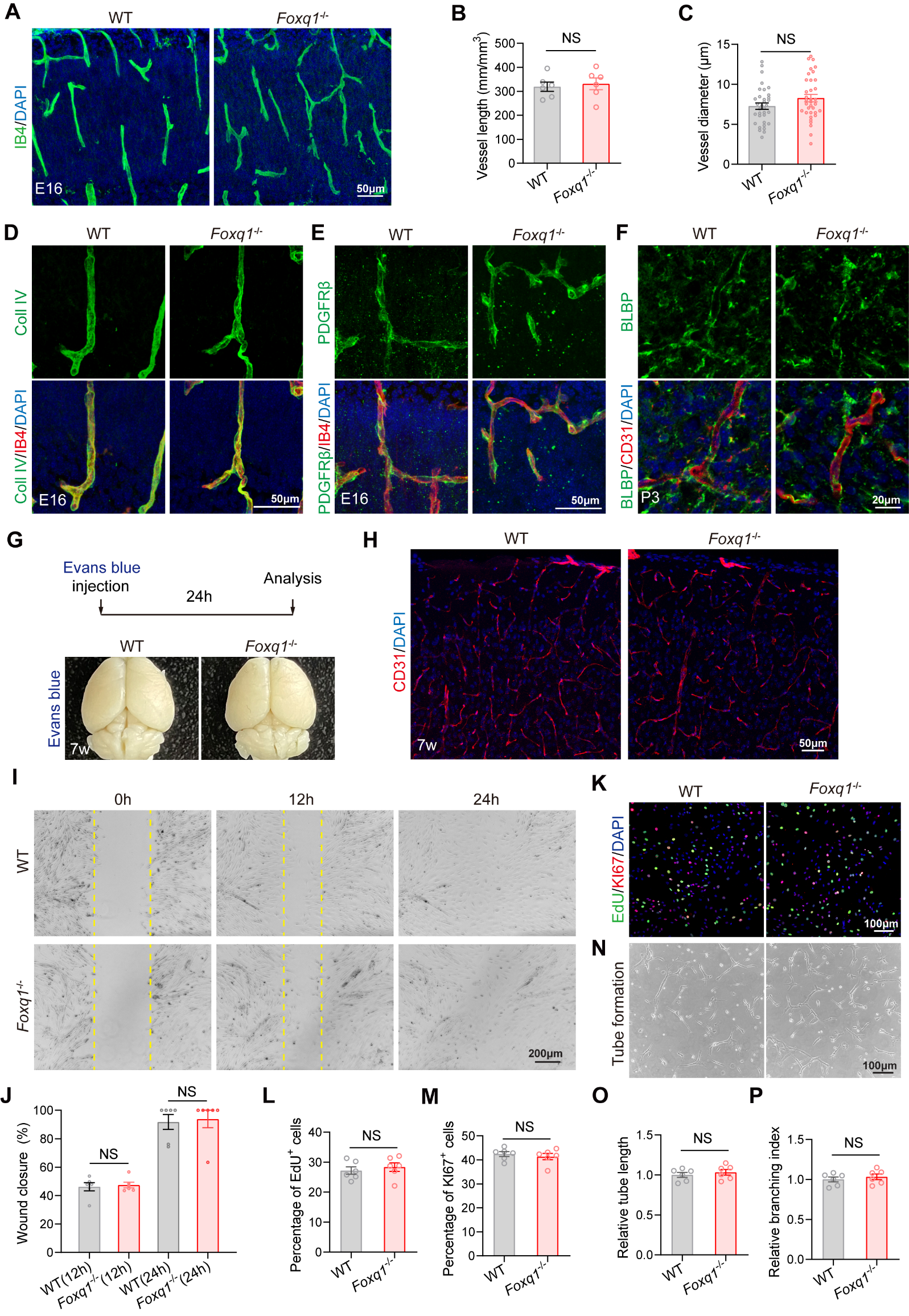


**Supplementary Figure 2.** FOXQ1 deletion preserves vascular development, blood-brain barrier integrity, and angiogenic functions. A) Representative images of E16 cortical sections from wild-type and *Foxq1* cKO mice staining with IB4 (green) and DAPI (blue). Scale bar, 50 µm. B) Quantification of the vessel length in wild-type and *Foxq1* cKO mice (*n* = 6 brains per group). C) Quantification of the vessel diameter in wild-type and *Foxq1* cKO cortical sections (*n* = 34 vessels in wild-type and 35 vessels in cKO). D) Immunofluorescence images of E16 wild type and *Foxq1* cKO cortex staining with IB4 (red), Collagen IV (green), and DAPI (blue). Scale bar, 50 µm. E) Immunofluorescence images of E16 wild type and *Foxq1* cKO cortex staining with IB4 (red), PDGFRß (pericyte maker) (green), and DAPI (blue). Scale bar, 50 µm. F) Immunofluorescence images of P3 wild type and *Foxq1* cKO cortex staining with BLBP (green), CD31 (red), and DAPI (blue). Scale bar, 20 µm. G) Representative whole-mount brain images of 7-week-old wild type and *Foxq1* cKO mice 24 hours post intraperitoneal injection of Evans blue dye. H) Immunofluorescence images of 7-week-old wild type and *Foxq1*^-/-^ cortex staining with CD31 (red) and DAPI (blue). Scale bar, 50 µm. I) Representative images from scratch wound assays measuring horizontal migration of wild-type and *Foxq1* cKO primary brain ECs. Scale bar, 200 μm. J) Quantification of wound closure rates at 12 and 24 hours (*n* = 6 replicates per group). K) Representative immunofluorescence images of wild-type and *Foxq1* cKO primary brain ECs following 4-hour EdU pulse labeling. Cells were stained for EdU (green), Ki67 (red), and nuclei with DAPI (blue). Scale bar, 100 μm. L,M) Quantification of EdU-positive and Ki67-positive cell percentages (*n* = 6 replicates per group). N) Representative images from tube formation assays using wild-type and *Foxq1* cKO primary brain ECs cultured on Matrigel for 12 hours. Scale bar, 100 μm. O,P) Quantification of relative tube length and branching points calculated using ImageJ software (*n* = 6 replicates per group). Data are shown as mean ± s.e.m for (B, C, J, L, O, and P). NS, no significant difference. Two-tailed unpaired student’s *t*-test.


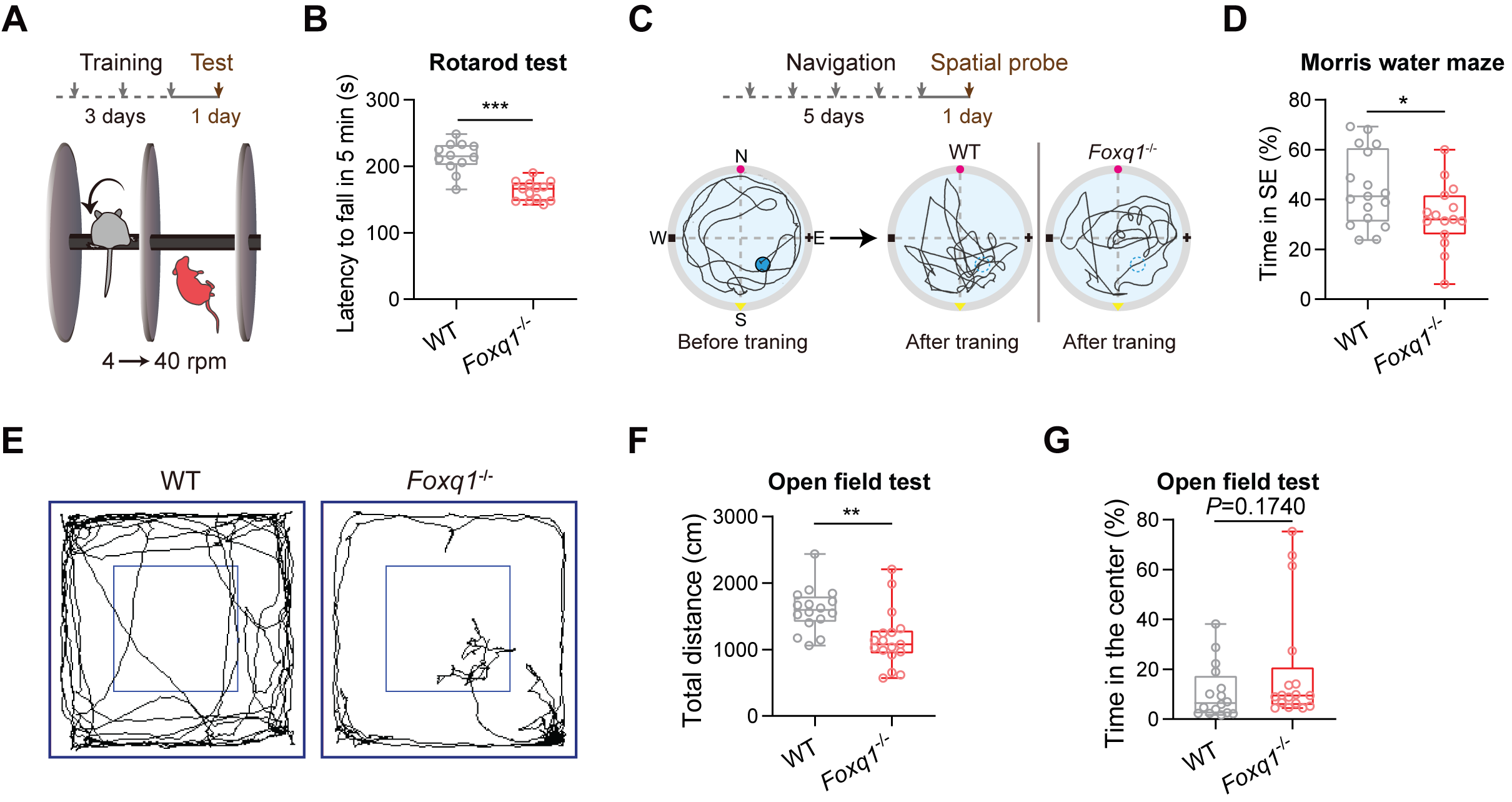


**Supplementary Figure 3.** *Foxq1* cKO mice displayed motor coordination deficits, impaired spatial memory, and altered exploratory behavior. A) Schematic for Rotarod test procedure. Wild-type and *Foxq1* cKO mice (12 and 13, respectively) underwent a 3-day training period with progressively increasing rod speeds. In the test session, mice were placed on a rotating rod accelerating from 4 to 40 rpm over 5 minutes to assess motor coordination and balance. B) Latency to fall in accelerating rotarod. The mean latency from three trials per mouse was used for analysis. C) Schematic for the Morris water maze procedure and representative swimming trajectories. Mice underwent 5 consecutive days of platform training, with four trials per day. During the probe trial, the platform was removed, and mice were allowed to swim freely for 60 seconds. D) Time spent in the target quadrant where the hidden platform was located, analyzed from 17 wild-type and 15 *Foxq1* cKO mice. E) Representative movement trajectories in the Open field test. Mice were placed in the center of a 50 cm × 50 cm arena and allowed to explore freely for 5 minutes. F,G) Total distance traveled and the time spent in the center zone (25 cm × 25 cm) during the 5-minute test were recorded for 16 wild-type and 17 *Foxq1* cKO mice.


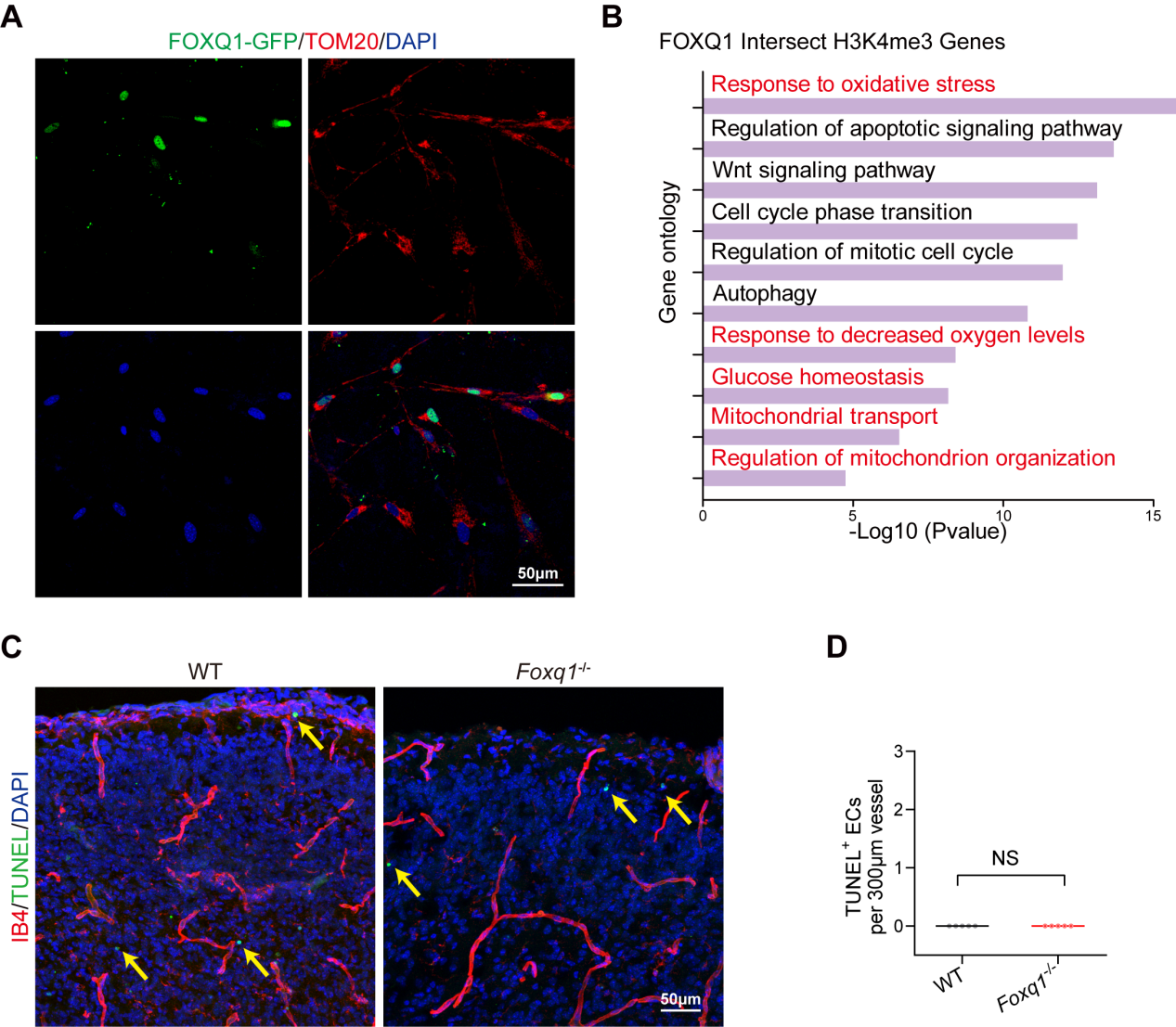


**Supplementary Figure 4.** FOXQ1 is localized in the nucleus and regulates the expression of mitochondria-associated genes in brain endothelial cells. A) Immunofluorescence images of primary ECs infected with the FOXQ1-GFP (green) lentivirus and stained with TOM20 (red) and DAPI (blue). Scale bar, 50 µm. B) Gene ontology (GO) enrichment analysis of the overlap of target genes between FOXQ1 and H3K4me3. Related to Figure 4C. C) Representative images of E16 wild type and *Foxq1* cKO cortex stained with IB4 (red), TUNEL (green), indicating apoptosis, and with DAPI (blue). Scale bar, 50 µm. D) Quantification of TUNEL-positive ECs per 300 µm of vessel length (*n* = 5 replicates from 2 brains). Data are shown as mean ± s.e.m. NS indicates not significant. Two-tailed unpaired Students’ *t*-test.

**
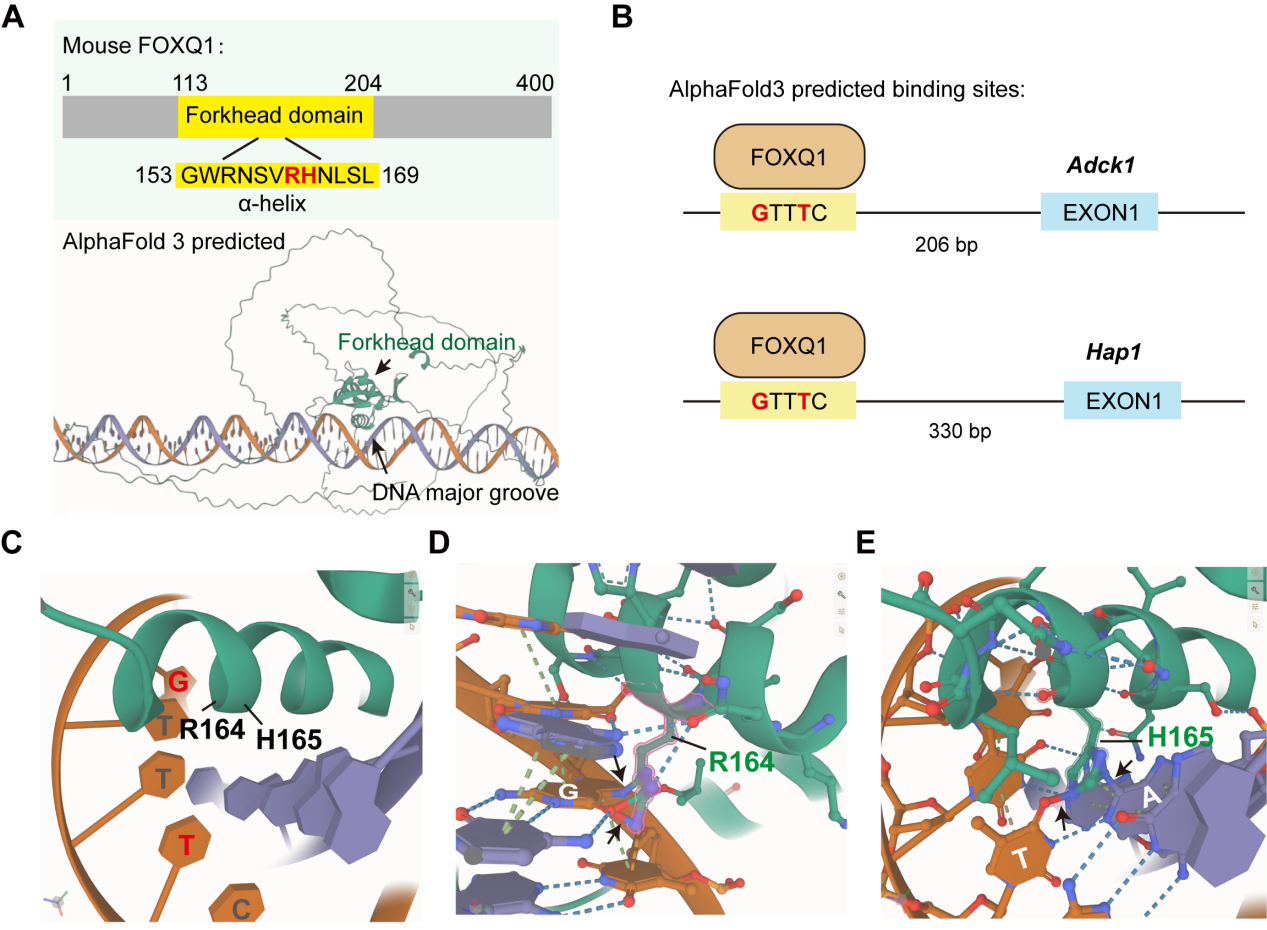
**

**Supplementary Figure 5.** Structural mechanism of FOXQ1 binding to DNA. A) (Top) Schematic of the domain organization of full-length mouse FOXQ1. (Bottom) The joint structure between FOXQ1 and DNA predicted by AlphaFold 3.^[22]^ The residues 153-169 of FOXQ1 form an α-helix structure that can insert into the major groove of DNA. Arginine (164) and histidine (165) residues are highlighted in red. B) Predicted binding sites of FOXQ1 at the mouse gene loci of *Adck1* and *Hap1*. The guanine highlighted in red can interact with arginine (164) residue, while the thymine highlighted in red can interact with the histidine (165) residue. C) Magnified view of the interaction between the α-helix and DNA major groove. D) Magnified view of the interaction between the guanidine group of arginine (164) and guanine. Red arrows indicate the new hydrogen bonds formed. E) Magnified view of the interaction between the imidazole group of histidine (165) and the A•T base pair. Red arrows indicate the new hydrogen bonds formed after the imidazole group inserts into the A•T base pair.

**
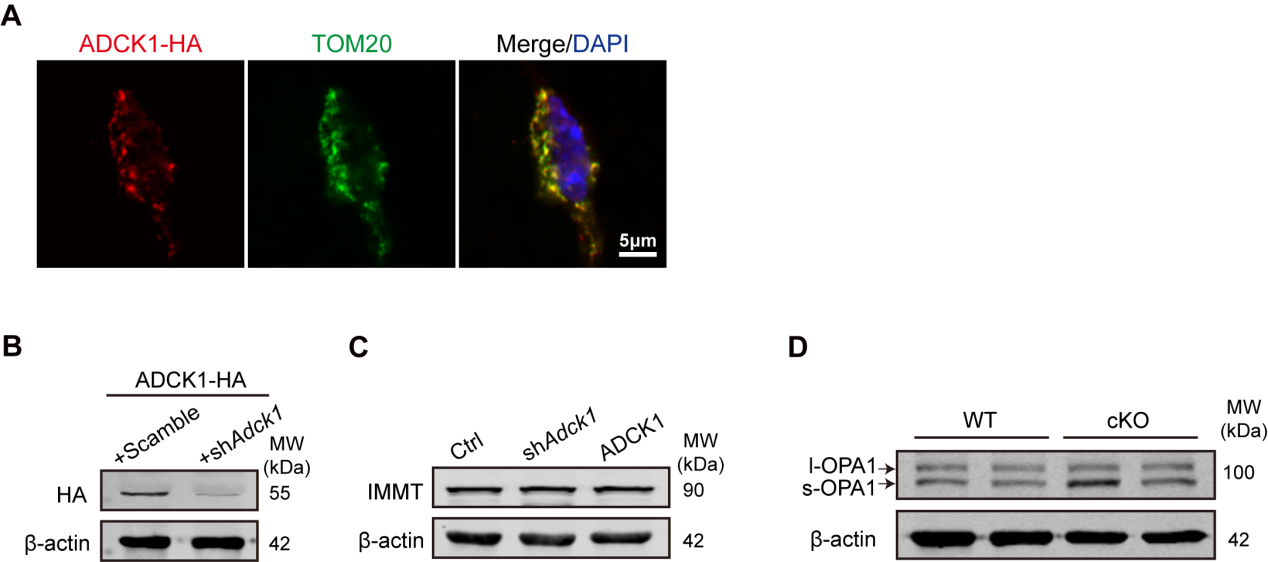


Supplementary Figure 6.** Cleavage of L-OPA1 in brain ECs following FOXQ1 deletion. A) Immunofluorescence images of primary brain ECs infected with *ADCK1-HA* (red) lentivirus, and stained with TOM20 (green) and DAPI (blue). Scale bar, 5 µm. B) Western blot analysis showing levels of HA-tagged ADCK1 in HEK293T cells transfected with either *Scramble shRNA* or *Adck1-shRNA1* to confirm knockdown efficiency. C) Immunoblot images for the expression of IMMT in primary brain ECs infected with either empty vector, *Adck1*-*shRNA*, or *ADCK1-HA* lentivirus. D) Immunoblotting images for the expression of OPA1 in primary brain ECs from both wild type and *Foxq1* cKO mice.

**Supplementary Table 1.** Gene Set Variation Analysis

| **Geneset** | **Brain** | **Liver** | **Lung** | **Kidney** | **Colon** |
| --- | --- | --- | --- | --- | --- |
| REACTOME_RECYCLING OF BILE ACIDS AND SALTS | 0.647 | -0.016 | 0.007 | 0.153 | -0.071 |
| REACTOME_AMINO ACID TRANSPORT ACROSS THE PLASMA MEMBRANE | 0.622 | -0.009 | -0.017 | -0.022 | 0.011 |
| GO_REGULATION OF CHOLESTEROL TRANSPORT | 0.470 | 0.052 | 0.065 | -0.026 | 0.133 |
| GO_OXYGEN TRANSPORT | 0.454 | 0.095 | 0.137 | 0.247 | 0.062 |
| GO_ORGANIC ACID TRANSMEMBRANE TRANSPORTER ACTIVITY | 0.444 | -0.140 | -0.174 | -0.234 | -0.097 |
| GO_POSITIVE REGULATION OF GLUCOSE METABOLIC PROCESS | 0.187 | 0.007 | -0.041 | 0.047 | -0.055 |
| MOOTHA_MITOCHONDRIA | 0.139 | 0.038 | -0.205 | -0.198 | -0.167 |
| REACTOME_TCA CYCLE AND RESPIRATORY ELECTRON TRANSPORT | 0.218 | -0.175 | -0.262 | -0.205 | -0.192 |
| GO_REGULATION OF COENZYME METABOLIC PROCESS | 0.078 | -0.160 | -0.248 | -0.108 | -0.167 |
| GO_POSITIVE REGULATION OF ATP METABOLIC PROCESS | 0.239 | 0.010 | -0.046 | 0.111 | -0.061 |
| GROSS_HYPOXIA VIA HIF1A DN | 0.196 | -0.295 | -0.059 | -0.439 | -0.078 |
| PID_HIF1 TFPATHWAY | 0.299 | -0.220 | -0.070 | -0.333 | -0.076 |
| WINTER_HYPOXIA UP | 0.254 | -0.180 | 0.147 | -0.040 | -0.077 |
| GO_REACTIVE OXYGEN SPECIES METABOLIC PROCESS | 0.170 | -0.012 | 0.003 | 0.030 | 0.011 |
| CHUANG_OXIDATIVE STRESS RESPONSE UP | 0.275 | 0.138 | 0.038 | -0.253 | -0.131 |
| GO_POSITIVE REGULATION OF OXIDOREDUCTASE ACTIVITY | 0.213 | 0.079 | -0.237 | -0.159 | -0.178 |
| WHITFIELD_CELL CYCLE M G1 | 0.159 | -0.076 | -0.079 | -0.100 | 0.012 |
| KAUFFMANN_DNA REPAIR GENES | 0.198 | -0.030 | 0.048 | -0.038 | -0.027 |
| REACTOME_METABOLISM OF RNA | 0.305 | 0.038 | -0.073 | -0.188 | -0.032 |
| BIOCARTA_NFKB PATHWAY | 0.358 | 0.115 | 0.195 | 0.071 | 0.082 |
| VILIMAS_NOTCH1 TARGETS DN | 0.288 | -0.122 | -0.081 | -0.109 | -0.140 |
| KARLSSON_TGFB1 TARGETS UP | 0.141 | -0.023 | -0.174 | -0.274 | -0.090 |
| KEGG_INOSITOL PHOSPHATE METABOLISM | 0.170 | -0.339 | -0.244 | -0.061 | -0.020 |
| GO_REGULATION OF NITRIC OXIDE SYNTHASE ACTIVITY | 0.097 | -0.013 | -0.038 | -0.085 | 0.058 |
| PID_ENDOTHELIN PATHWAY | 0.237 | -0.049 | 0.205 | -0.081 | 0.034 |
| REACTOME_CELL SURFACE INTERACTIONS AT THE VASCULAR WALL | 0.243 | -0.103 | 0.099 | -0.121 | 0.176 |

**Supplementary Table 2.** Primers for PCR and qRT-PCR.

| HAP1-F | ATGCGCCCGAAAGAGCAG |
| --- | --- |
| HAP1-R | ATATGGTTGATGATCGAAAGCTTGT |
| IP3R1-F | ACCTTTGAAGAGCACATCAAGG |
| IP3R1-R | GGCCGGCTGCTGTGG |
| ADCK1-F | ATGGCCAGAAAGGCTCTCAAG |
| ADCK1-R | CATTCTGTGTGGAGCCCGA |
| OPA1-F | ATGTGGCGAGCAGGTCGG |
| OPA1-R | CTTCTCCTGGTGAAGAGCTTCAAT |
| FOXQ1-F | AAATTGGAGGTGTTCGTCCC |
| FOXQ1-R | TCAAGCTAGCAGAGTCTCCACC |
| HAP1-RT-F | GAGGTGGACGAGACCACAAG |
| HAP1-RT-R | TGATCGAAAGCTTGTCCCACT |
| BNIP3-RT-F | AACAGCACTCTGTCTGAGGAAG |
| BNIP3-RT-R | TTCCCCCTTTCTTCATAACGCT |
| OMA1-RT-F | GCCGAAGCTGACAAAGTTGG |
| OMA1-RT-R | AGACCTCCCGGAGTTTGAGA |
| ADCK1-RT-F | TGGCCAGAAAGGCTCTCAAG |
| ADCK1-RT-R | TGGGATCGACGCTGCAAATA |
